# Harnessing multi-omics approaches to combat Karnal bunt of wheat: a review of advances and future prospects

**DOI:** 10.3389/fgene.2025.1687301

**Published:** 2025-10-06

**Authors:** Renu Sharma, Satish Kumar, C. N Mishra, O. P. Ahlawat, Ratan Tiwari

**Affiliations:** ICAR – Indian Institute of Wheat and Barley Research, Karnal, India

**Keywords:** wheat, Karnal bunt, multi-omics, disease resistance, machine learning

## Abstract

Karnal bunt of wheat, caused by the fungus *Tilletia indica*, is a major quarantine disease that not only affects global wheat trade but also leads to yield loss and reduced grain quality. With global climate change, the disease has spread to new areas across continents, increasing vulnerabilities and creating a worrisome scenario, as once established, it is extremely difficult to eradicate. Host resistance remains the most effective strategy to combat Karnal bunt. However, only a few resistant sources have been identified so far and are being deployed in breeding programs. Various omics approaches including genomics, transcriptomics, proteomics and metabolomics have gained considerable attention for their role in enhancing disease resistance and improving agronomic yield in wheat. Notably, the integration of multiple omics and epiomics strategies has led to substantial advancements in identifying candidate genes, analyzing pathways, and understanding key elements of stress responses, thereby improving yields. Renowned for its data-mining capabilities, Machine Learning offers an opportunity to enhance the precision of current trait association methods. Nonetheless, its application in predicting disease resistance is still not widespread. In this review, we explore various omics technologies and platforms employed in wheat research to deepen the understanding of the molecular mechanisms involved in host-pathogen interactions, thereby advancing resistance to Karnal bunt of wheat. Furthermore, we emphasize the potential of Machine Learning as a significant tool for pinpointing genetic loci that contribute to host resistance.

## Introduction

Wheat is a crucial crop ensuring global food and nutritional security. Despite its high productivity, it is threatened to numerous biotic and abiotic challenges. Among the biotic stresses, fungal diseases such as rusts and smuts are the most significant threats to wheat. Karnal bunt, caused by the hemi-biotrophic fungus *T. indica* (syn. *Neovossia indica*), is considered a “minor agronomic but major quarantine threat,” highlighting its dual impact on crop quality and global wheat trade dynamics. First reported in 1931 in the Karnal district of India (Mitra, 1931), it has since spread to major wheat-growing regions in India and other countries (Bonde et al., 2004a). The pathogen is recognized as an international quarantine fungal pathogen, with the disease reported in countries like Afghanistan, Pakistan, Nepal, Mexico, parts of the United States, Iraq, Iran, Lebanon, Syria, Sweden, Turkey, and South Africa. Currently, it poses a significant biosecurity concern for wheat exports (Warham, 1987; Tan et al., 2013; Bishnoi et al., 2020). Due to Karnal bunt spore contamination, wheat exports from India have been rejected in Turkey and numerous European countries, resulting in stringent quarantine regulations and export prohibitions (Bishnoi et al., 2020). Similar to this, several importing nations rejected wheat imports after KB was found in US states like Arizona, which disrupted commerce and resulted in financial losses. (Utah Department of Agriculture and Food, 1996). Climate change conditions in the coming years have raised warnings about the disease (Gurjar et al., 2016). *Tilletia indica* is a soil, seed, and air-borne fungus that primarily infects the floral parts of wheat (Bains and Dhaliwal, 1989; Carris et al., 2006). Identifying Karnal bunt disease in the field is challenging due to its subtle symptoms. The distinctive symptom is the development of bunt sori on only a few grains in the head, rather than the entire head (Dhaliwal et al., 1988). Another notable symptom is the rotten fishy smell emitted by infected grains, caused by the presence of the trimethylamine compound (Mitra, 1937). Seed- or soil-borne teliospores appear to initiate Karnal bunt infection (Dhaliwal, 1989; Goates, 2010). The thick-walled, resilient teliospores of *T. indica* can travel long distances and persist as seed contaminants. Teliospores of *T. indica* can travel over long distances and persist as contaminants in seeds. Contamination levels exceeding 1% degrade wheat quality due to a fishy odor and black discoloration. When more than 3% of seeds are infected, the wheat becomes unsuitable for human consumption (Warham, 1990), leading to economic losses. The disease poses a threat to countries free of the pathogen, as undetected teliospores can gradually establish themselves. Nations such as Australia, Canada, and the United States enforce a zero-tolerance policy for Karnal bunt spores in wheat imports to prevent the pathogen’s establishment (Singh J. et al., 2020; Kumar et al., 2021).

**FIGURE 1:**
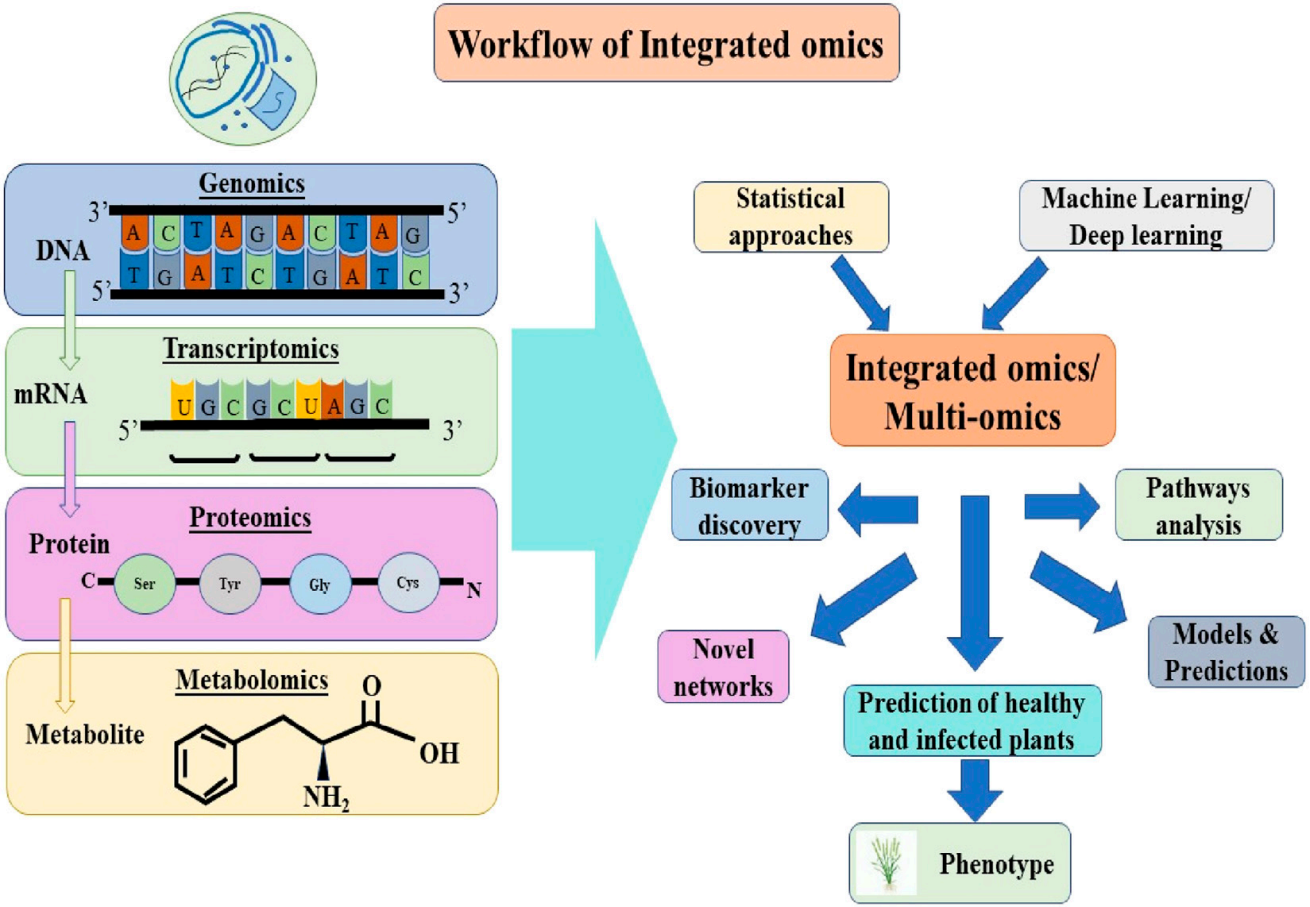
Schematic workflow of multi-omics approaches and AI in plant disease resistance in wheat.

Consequently, it is crucial for wheat breeding initiatives in the impacted and at-risk nations to intensify their focus on identifying and developing resistant varieties. In comparison to rusts and mildews, the progress in identifying, characterizing, and cloning KB resistance genes has been significantly slower (Singh S. et al., 2020; Bishnoi et al., 2020). Recent progress in omics technologies, including genomics, transcriptomics, proteomics, and metabolomics, has facilitated extensive research aimed at unraveling the mechanisms of stress tolerance. This has led to a deeper understanding of gene expression, protein profiling, and the biological processes that contribute to various stress tolerance traits (Ma et al., 2022). To manage the vast amount of data produced by these omics approaches, new analytical tools, high-throughput data analysis pipelines, and omics databases have been established (Yuan et al., 2017; Ma et al., 2021; Zhang et al., 2021). All these approaches offer a deeper understanding of the complex regulatory networks that govern cellular functions and pathways. In recent years, multi-omics has gained prominence as a key strategy for deciphering the plant’s response to abiotic and biotic stresses and for building predictive models thereby enabling extensive research in various crops, including wheat as depicted in Figure 1. In contemporary plant breeding, leveraging cutting-edge technologies to boost disease resistance is of paramount importance. Renowned for its prowess in data mining, Machine Learning presents a chance to enhance the precision of current trait association techniques and has been utilized to forecast a range of agronomic traits across different species. This review underscores the advent of advanced next-generation sequencing techniques and computational technologies, multi-omics approaches and tools in combination with the potential of AI and various Machine Learning models paving the way for enhanced resistance to Karnal bunt of wheat, fostering sustainable agriculture, and stress resilience, thereby bolstering food security.

## Epidemiology of Karnal bunt

The KB pathogen *T. indica* can be transmitted through soil, seeds, or air, with its occurrence influenced by favorable environmental conditions (Kashyap et al., 2011; Biswas et al., 2013). A cold and humid climate promotes KB infection *via* teliospores, which attack the wheat grain pericarp. Carris et al. (2006) reviewed that teliospores are robust, with a high capacity for survival even in harsh conditions, showing resistance to toxic gases, chemicals, and low pH levels. These teliospores can endure in desert and frosty environments for extended periods, with a viability of up to 5 years under extreme stress (Bonde et al., 2004b). Teliospores typically undergo a dormancy period of 1–6 months before germinating (Prescott, 1984), with the highest germination rates observed in teliospores that are a year old (Bansal et al., 1983). This dormancy feature enhances the survival of *T. indica*. Once dormancy ends, teliospores germinate on the soil surface under suitable conditions to infect wheat plants. Understanding the genetic control of teliospore dormancy could aid in developing a race designation system for *T. indica*. Conditions of low precipitation combined with high humidity and cold temperatures (8 °C–20 °C) are ideal for teliospore germination (Duveiller and Mezzalama, 2009). However, KB outbreaks remain unpredictable, as infections may not occur even when conditions and inoculum are favorable. Prioritizing the understanding of pathogen-environment interactions is essential for accurate disease forecasting.

The development of *T. indica* commences during the liberation of teliospores from infected spikes while harvesting and are dispersed by the wind, leading to KB in the following season. These teliospores germinate to form primary sporidia in the subsequent crop cycle. Stubble burning facilitates the long-distance travel of teliospores, allowing them to move up to 3 km from the plot (Bonde et al., 1987), which is a critical consideration in wheat production and stubble management. Although teliospores can be carried by the wind and survive digestion by animals, the disease mainly spreads internationally through contaminated seeds (Duveiller and Mezzalama, 2009). The disease’s significant spread potential is due to its small, resilient teliospores being dispersed by wind, seeds, containers, machinery, humans, birds, and animals. The germinated teliospores (allantoid sporidia) are carried to the flag leaf by wind or rain, where they multiply and infect spikelets through rainwater or dew (Carris et al., 2006). Research on the longevity of allantoid sporidia has yielded varying findings. Initial studies by Aujla et al. (1985) and Nagarajan et al. (1997) suggested that these sporidia have a short lifespan due to drying out. In contrast, subsequent research found that they could remain viable for up to 60 days at 40%–50% relative humidity and 18 °C and for more than 46 days at temperatures above 40 °C with 10% relative humidity (Goates and Jackson, 2006). Goates (2010) discovered that sporidia can stay dormant in dry soil and quickly regenerate when exposed to moisture. Although germination has little impact on spore durability, the viability of germinated secondary sporidia declines when relative humidity drops below 76% and temperatures exceed 24 °C (Biswas et al., 2013). The infection by *T. indica* spreads from the rachis to the glumes during the flowering and grain development stages, with fungal hyphae invading the germinal end of developing seeds (Riccioni et al., 2008). Once infection is established, teliospores develop in the middle layers of the seed pericarp, leading to endosperm contraction and layer splitting (Carris et al., 2006), eventually being replaced by teliospore powder. The embryo usually remains viable except in severe cases, although significant damage to the endosperm can occur (Fuentes-Davila et al., 1996). Severe damage to the embryo can prevent germination, thereby reducing plant growth and yield. During harvest, teliospores are dispersed through air and soil, potentially causing new infections under suitable conditions in the following season (Kumar and Nagarajan, 1998). Table 1 summarizes the environmental factors favoring KB infection.

**TABLE 1:** Environmental factors favoring KB infection.

S. No.	Environmental factor	Description	Key points/Effects	References
1	Temperature	Optimal range 8 °C–20 °C (cold climate)	Favors teliospore germination and infection	Duveiller and Mezzalama (2009)
2	Humidity	High relative humidity ≥70%; wet conditions during heading	Prolonged wetness favors sporidia germination and infection	Carris et al. (2006), USDA APHIS (2007)
3	Dormancy	Teliospores dormant 1–6 months; max germination at 1 year	Ensures long-term survival and delayed infection	Prescott (1984), Bansal et al. (1983)
4	Teliospore resilience	Survive harsh environments (desert, frost), resistant to chemicals, gases, low pH	Teliospores survive years in soil and stored seed	Bonde et al. (2004a), Carris et al. (2006)
5	Dispersal	Wind disperses spores and sporidia; stubble burning aids spread up to 3 km	Facilitates local spread; international spread mainly *via* contaminated seed	Bonde et al. (1987), Duveiller and Mezzalama (2009)
6	Sporidia longevity	Allantoid sporidia can remain viable days to weeks under moderate humidity and temperature	Long viability enhances infection potential	Goates and Jackson (2006), Biswas et al. (2013)
7	Infection conditions	Infection occurs during flowering and grain development under cold and humid microclimate	Infection of spikelets, seed pericarp colonization	Riccioni et al. (2008), Carris et al. (2006)
8	Environmental unpredictability	Outbreaks unpredictable despite favorable conditions and inoculum presence	Emphasizes need for better pathogen-environment interaction understanding	Biswas et al. (2013)

## Genetics of host resistance mechanisms to *Tilletia indica* infection

Hemibiotrophs like *T. indica* (KB pathogen) initially establish a silent, symptomless infection and suppress host defenses, making early detection difficult and allowing the pathogen to escape many defense responses that are effective against biotrophs (such as HR). The pathogen later shifts to necrotrophy, killing host tissue and spreading as a necrotroph. This phase exposes additional challenges, since conventional necrotroph management (e.g., fungicides, debris management) may be too late or less effective due to deep tissue infection and systemic spread. The pathogen’s hemibiotrophic lifecycle enables it to evade typical resistance mechanisms, complicating genetic control. Asymptomatic, latent infections facilitate undetected spread and make quarantine enforcement difficult. The complex, quantitative nature of resistance and strong genotype-by-environment effects further hinder reliable breeding and epidemiological management (Brar et al., 2018; Bishnoi et al., 2020).

Resistance to KB is revealed *via* both morphological and physiological traits. Early studies (Warham, 1988) emphasized that triticale and durum wheat are generally more resistant than bread wheat, largely due to morphological traits like pubescence and compact spike structure, which physically impede fungal invasion. However, the contribution of these morphological traits is not absolute. For example, Kumar and Nagarajan (1998) demonstrated that the posture of the flag leaf can influence KB infection, as a sharp angle between the flag leaf and the boot may funnel allantoid sporidia toward the spike, potentially increasing susceptibility. Gogoi et al. (2002) showed KB susceptible cultivars like WL711 typically have more stomata and lower hair counts than resistant genotypes, suggesting stomatal density and hairiness on glumes and rachis may act as barriers. In addition, resistant lines were characterized by compact spikelets and narrower glume openings, potentially reducing pathogen entry. Yet, Aujla et al. (1990) and Singh (1992) provided contrasting results regarding spikelet compactness as a resistance factor—whereas Aujla et al. associated compact spikelets with resistance, Singh did not find a significant effect under artificial inoculation, likely because this method bypasses natural barriers by directly injecting teliospores into the plant structure.

Collectively, these findings suggest that while morphological barriers such as pubescence, spikelet compactness, and glume structure can enhance resistance, their effectiveness is context-dependent. Under natural conditions, these traits may block or delay infection, offering a degree of field resistance or escape. However, under artificial inoculation or high disease pressure—when the pathogen bypasses external barriers—these morphological traits alone may not be sufficient to confer resistance. Additionally, early anthesis may serve as an escape mechanism, but this too is influenced by environmental factors and the timing of pathogen attack. Therefore, reliance solely on morphological defense traits could lead to inconsistent resistance expression, underscoring the need to combine morphological, physiological, and genetic resistance strategies in breeding programs.

Resistance to KB in the host is inherited quantitatively, with numerous loci of minor effect contributing additively to resistance on a continuous scale (Fuentes-Davila et al., 1995; Nelson et al., 1998; Singh et al., 2003; Singh et al., 2007). Resistance to KB is dominant or partially dominant over susceptibility (Singh et al., 1995b; Villareal et al., 1995), with several genes exhibiting dominant, duplicate dominant, and complementary gene actions (Morgunov et al., 1993; Fuentes-Davila et al., 1995; Singh et al., 1999; Tyagi et al., 2010). The number of genes that determine KB resistance and their interactions, whether they are dominant or recessive, varies across genotypes. Most genetic studies have pinpointed between one and six key resistance genes (Morgunov et al., 1993; Fuentes-Davila et al., 1995; Singh et al., 1995a; Singh et al., 1995b; Singh et al., 1999; Swati and Goel, 2010). However, Sharma et al. (2005) identified up to nine loci associated with KB resistance in “HD29,” “W485,” “ALDAN'S”/”IAS58,” and “H567.71/3∗PAR,” with the genetic variability of the parental genotypes not explaining the observed differences. Fuentes-Davila et al. (1995) identified nine loci with non-allelic genes across four resistant parents. KB resistance is governed by a single recessive gene (Bag et al., 1999), and two or three additive genes (Sehgal, 2006) and two or more additive genes (Sirari et al., 2008) in various genotypes. Virdi et al. (2016) identified that a single recessive gene governs KB resistance in “W8627 × PBW343” populations, concluding that this resistance can be effectively managed in segregating generations. The pre-breeding/genetic characterization aspect remains vital in KB resistance breeding. High heritability estimates are crucial for the transmission of traits to subsequent generations. The heritability estimates reported for KB resistance suggest a strong genetic basis (Gupta et al., 2019), indicating that KB resistance is suitable for QTL mapping. Brar et al. (2018) reported higher heritability values of 0.75 and 0.78 in wheat populations, while Emebiri et al. (2019) reported a value of 0.69. Emebiri et al. (2019) linked the high heritability observed in KB genetic studies to the precise phenotypic screening methods developed by Fuentes-Davila et al. (1995). These protocols effectively minimize environmental influences during field screening, enabling accurate measurement of genetic inheritance. The high heritability estimates indicate that KB resistance in wheat is highly heritable and governed by “relatively simple” genetics, although further validation through genetic studies is still necessary.

## Genomics

The technique of uncovering the chromosomal regions controlling such complex traits in plants and detecting the closely linked markers is known as QTL mapping. These maps can be utilized in breeding for QTL analysis and MAS (Kaur et al., 2015; Manickavelu et al., 2011). Traditionally, QTL identification for KB resistance in wheat has relied on biparental populations, primarily RILs. QTL with significant effects on KB resistance are uncommon, possibly due to limited parental variability or environmental factors that obscure genetic effects. Although large-effect QTL are easier to detect, complex traits generally exhibit average QTL effects (Mackay, 2001). The current understanding, based on a few major QTL, remains inadequate. The most significant QTL, accounting for 25% of phenotypic variance, is located on the 4BS chromosome in “HD29” and is associated with “Xgwm538” (Singh et al., 2003), which was later converted to an SNP marker by Brooks et al. (2006). The QTL “*Qkb.ksu 5BL.1*” on chromosome 5BL explained 19% of the variance, while “*Qkb.ksu 6BS.1*” on 6BS accounted for 13% (Table 2). Studies have documented QTL with minor effects (Bishnoi et al., 2020). To eliminate bias in detected KB QTL, it is necessary to compare them with expected values to identify any loci that may have been overlooked (Myles and Wayne, 2008). Gupta et al. (2019) identified 18 genomic regions explaining 5%–20% of the variation and one consistent QTL on 2BL in Afghan wheat accessions. The phenotypic variation attributed to major effect QTL might be overestimated due to small sample sizes, a phenomenon known as the “Beavis effect” (Beavis, 1994). Functional genomics and ESTs can help identify tightly linked markers.

**TABLE 2:** QTLs identified in previous studies for resistance to KB in wheat.

Sr. No.	Line/genotype/origin	Chromosome	Linked marker/Interval/physical position	References
1	Altar 84	3BS, 5AL	RFLP	Nelson et al. (1998)
2	HD29	4BL	Xgwm538	Singh et al. (2003)
3	HD29	4BL	(gwm538 SNP)	Brooks et al. (2006)
4	HD29	*Qkb.ksu-5BL.1*	Xgdm116-Xwmc235	Singh et al. (2007)
5	HD29	*Qkb.ksu-6BS.1*	Xwmc105-Xgwm88	
6	W485	*Qkb.ksu-4BL.1*	Xgwm6-Xwmc349	
7	H567.71	4B	Xgwm6	Kumar et al. (2015)
8	ALDAN	*Qkb.dwr-5BL.1*	Xwmc235-Xbarc140	Kaur et al. (2015)
9	HD29	5B	Xgdm116-Xwmc235	
10	HD29	6B	Xwmc105-Xgwm88	
11	W485	4B	Xgwm6-Xwmc349	Bala et al. (2016)
12	WKCBW	*Qkb.cim-2BL*	1086228–1092041	Brar et al. (2018)
13	WKCBW	*Qkb.cim-3DL*	7487658–2252,592	
14	Huirivis#1	*Qkb.cim-3BS1*	1079551–100010977	
15	Mutus	*Qkb.cim-5BS2*	2253589–1011847	
16	HD29	*Qkb.cim-2BL*	IWB57185	Emebiri et al. (2019)
17	WH542	*Qkb.cim-2BL*	IWA1644	
18	WH542	21D	IWB2650	
19	W485	1B	B59865	
20	Afghanistan panel	1DL	470084827	Gupta et al. (2019)
		2DL	586853396	
		4AL	656758037	
		5AS	36718388	
		6BL	500595153	
		6BS	21209894	
		7BS	45306426	
		7DL	607297738	

Advancements in next-generation sequencing (NGS) technologies have been instrumental in scientific discoveries, offering genomic tools that enhance wheat research and transform breeding methods (Hussain et al., 2022). In the field of wheat, efficient genotyping platforms can be integrated with physical maps to facilitate gene discovery (Rasheed et al., 2017). SNP genotyping arrays now enable the rapid and cost-effective screening of thousands of markers within populations. Various SNP arrays have proven the successful genotyping of wheat. The 9K iSelect array assessed 2,994 wheat accessions, while the development of a 15K Infinium array was spurred by a 90K SNP chip. The Affymetrix Axiom 820K array identified polymorphisms in bread wheat, which led to the creation of a 35K Wheat Breeders Array. Resequencing data from eight wheat lines resulted in a 280K array for related accessions. Additionally, a 660K Axiom array was designed based on previous arrays, and an Illumina 40K array captures the diversity of both wheat and barley (Sun et al., 2020). NGS-based genotyping-by-sequencing detects variation in wheat germplasm. The presence of millions of SNPs in wheat has significantly advanced the discovery of new genes through genome-wide association studies (GWAS). The use of GWAS analysis in identifying wheat KB resistance is still relatively underutilized. Over 3,000 marker-trait association related to agronomic traits in wheat have been consolidated into 141 meta-QTLs, with thirteen identified as breeder’s meta-QTLs for yield improvement.

Marker-assisted selection (MAS) in both structured and unstructured families has successfully addressed the challenges of field screening and improved the precision of identifying resistance to KB, as reported by Kumar et al. (2016). Traditional breeding methods face limitations because selecting for minor KB resistance genes is challenging due to partial resistance and additive gene effects (Bala et al., 2016). Genome-wide association studies (GWAS) in unstructured germplasm panels facilitate the creation of biparental populations. Gupta et al. (2019) discovered QTLs on chromosomes 1DL, 2DL, 4AL, 5AS, 6BL, 6BS, 7BS, and 7DL. Emebiri et al. (2019) identified two notable clusters on chromosome 4B (*Qkb.ksu-4B*, *QKb.cimmyt-4BL*, *Qkb.cim-4BL*) and chromosome 3B (*Qkb.cnl-3B*, *QKb.cimmyt-3BS*, *Qkb.cim-3BS1*). Due to the possibility of false positives from small panel sizes, GWAS analysis requires validation.

Conventional genome-wide association studies (GWAS) face challenges in detecting rare variants, particularly with complex gene compositions like haplotypes. Haplotype-based GWAS improves statistical power for marker-trait associations, enables better delineation of candidate regions, and captures combinatorial effects of linked variants, providing a framework for analyzing quantitative traits in crops (Qian et al., 2017). In 2022, Hamazaki and colleagues introduced “RAINBOW,” a SNP-set method for haplotype-based genome-wide association studies (GWAS) that utilizes haplotype blocks without the need for pre-existing haplotype data. They evaluated using 100 simulated phenotypic datasets with *Oryza sativa* marker genotype data and comparing it to single-SNP GWAS, haplotype-based GWAS, and SNP-set GWAS. The study demonstrates that SNP-set GWAS offers superior sensitivity not only for detecting rare variants but also for identifying genes characterized by complex genetic architectures, such as those harboring multiple causal variants. Radecka-Janusik et al. (2022) utilized haplotype-based GWAS to pinpoint chromosomal regions in derived wheat families associated with resistance to Fusarium head blight. The robust data set and the substantial proportion of phenotypic variance explained by the marker-trait associations provide a promising foundation for the application of these findings in marker-assisted selection (MAS). Lisker et al. (2022) performed a haplotype-based GWAS study and identified trait-improving QTL alleles controlling agronomic traits under contrasting nitrogen fertilization treatments in the magic wheat population WM-800. Koua et al., 2024 Genome-wide dissection and haplotype analysis identified candidate loci for nitrogen use efficiency under drought conditions in winter wheat.

Understanding wheat genes and genomic elements is essential for genetic enhancement. Several reference genome sequences have been published for wheat varieties, including durum wheat, bread wheat, and progenitor species. These assemblies have unveiled the genomic landscape of wheat and enabled genome-wide analysis of repeat and gene families, revolutionizing wheat genomics. Multiple reference genomes and resequencing data have demonstrated significant genomic variation. The recognition that a single reference genome is insufficient to fully represent species diversity has led to the advent of wheat pan-genomics. Pan-genomes delve into the complete spectrum of sequence diversity within a species, covering both core (universal) and unique (specific) genomic elements, which are essential for understanding agronomic traits. The initial wheat pan-genome study, which examined 18 different cultivars, discovered new gene regions by analyzing presence and absence variations in comparison to the Chinese Spring reference genome (Montenegro et al., 2017; Tiwari et al., 2024), highlighted the existence of over 50 reference-level genome assemblies, while (Bayer et al., 2022), pioneered the development of the first wheat graph pangenome using 16 cultivars with Panache visualization. Long-read sequencing facilitates complete genome assemblies, offering insights into complex loci and centromere diversity. Functional annotation of these variable gene components revealed a wealth of stress-response genes. Dispensable gene sets showed greater genomic variation than core genes, contributing to crop diversity. Reference genome assemblies enabled the *in silico* identification of gene families with stress-related functions. Nucleotide-binding leucine-rich repeats (NLR) gene family exploration revealed diverse resistance gene patterns, displaying only 30%–34% of NLR signatures conserved across the different lines, providing valuable variation data for breeding disease-resistant cultivars. Given the 16 Gb size of the wheat genome, the wheat pangenome will be one of the largest among crop plants, requiring significant computing infrastructure that the wheat research community must prepare for. An international consortium is expanding the wheat pangenome by incorporating genomic data from a diverse array of global genotypes (Thudi et al., 2025). This collaborative effort is poised to accelerate wheat improvement by leveraging advanced genomics and enabling the discovery of novel genetic variation for breeding. Investigating gene families allows for the identification of members, structural analysis, evolutionary relationships, expression dynamics, and functional assignments. Numerous studies have explored the functional roles of gene families under stress conditions in wheat.

In the hexaploid genome of wheat, KB resistance exhibits unique inheritance patterns. Although *Ae. tauschii* (the D-genome donor) shows KB resistance, the D-genome is less polymorphic compared to the highly polymorphic B-genome. This highlights the need to scan the D-genome using diverse *Triticum aestivum* lines. Singh S. et al. (2020) identified candidate genes on chromosome 4D, indicating the D-genome’s potential for KB resistance. Non-pleiotropic KB resistance genes may not have been favored by natural selection due to the absence of the pathogen, making Indian wheat germplasm an important area for study. Future research on KB should aim to enhance the resolution of previously mapped QTL and discover new ones. Understanding QTL with small effects is essential for developing comprehensive genetic models of KB resistance (Lorenz and Cohen, 2012). Genomic selection (GS) stands out for its remarkable ability to accelerate genetic progress by enhancing selection accuracy while simultaneously reducing breeding time and costs. The current high density of markers facilitates haplotype-based genome-wide association studies (GWAS), analyses of epistatic interactions, GS, and selective sweep analyses.

This integration of genomics has revolutionized breeding programs worldwide, improving marker-assisted selection (MAS) and GS for more rapid breeding outcomes. Research has utilized GS for abiotic stress tolerance and assessed statistical models to enhance prediction accuracy. Efforts have been directed towards optimizing genomic prediction accuracies for resistance to wheat pathogens, such as powdery mildew (*Blumeria graminis*), fusarium head blight (*Fusarium graminearum*), septoria tritici blotch (*Zymoseptoria tritici*), stem rust (*Puccinia graminis* Pers), leaf rust (*Puccinia triticina* Eriks), stripe rust (*Puccinia striiformis* West), stagonospora nodorum blotch (*Parastagonospora nodorum*), spot blotch (*Bipolaris sorokiniana*), and tan spot (*Pyrenophora tritici*-repentis). Developing wheat varieties that are both climate-resilient and high-yielding necessitates the integration of omics data with genomic selection, high-throughput phenotyping, and gene editing.

## Transcriptomics

The central dogma of molecular biology outlines the process by which genetic information is stored in DNA, transcribed into RNA, and subsequently translated into proteins (Crick, 1970). This genetic information, shaped by environmental factors, dictates an organism’s phenotype. The transcription of genes into RNA molecules is pivotal in defining cell identity and regulating biological functions. These RNA molecules, collectively known as the transcriptome, are essential for interpreting genome function and gaining insights into development and disease. Early research on gene expression employed techniques such as northern blots and quantitative polymerase chain reaction (qPCR) to analyze individual transcripts. However, advancements have led to the development of transcriptomics, which allows for genome-wide measurement of gene expression. Challenges in this field include the necessity for prior sequence knowledge, issues with cross-hybridization artifacts, and difficulties in quantifying extreme expression levels (Casneuf et al., 2007; Shendure, 2008). Sequence-based methods were introduced to directly determine transcript sequences. Expressed sequence tag (EST) libraries were created through Sanger sequencing of complementary DNA (cDNA), though this method had limited throughput (Adams et al., 1991). Tag-based methods like serial analysis of gene expression (SAGE) and cap analysis gene expression (CAGE) provided higher throughput and precise quantification by counting tagged sequences (Velculescu et al., 1995). Nevertheless, these methods were unable to detect splice isoforms or discover new genes and were constrained by cloning requirements and costs. The advent of high-throughput next-generation sequencing (NGS) revolutionized transcriptomics through RNA sequencing (RNA-Seq), enabling comprehensive analysis of gene expression, alternative splicing, and allele-specific expression (Wang et al., 2009).

Recent developments in RNA-Seq workflow from sample preparation to sequencing platforms to bioinformatic data analysis, have made it possible to deeply profile the transcriptome and provide insight into a variety of physiological and pathological states. Research on wheat has made substantial use of RNA-Seq. According to latest reports, RNA-Seq global transcriptome profiling analysis can detect alternatively spliced isoforms, coding genes, and differentially expressed lncRNAs in response pathogen infection. The role of various TFs belonging to the NAC, WRKY and MADS families was highlighted to be a significant one under a single or multiple abiotic stress condition while TFs from the MYB family were highlighted as the key candidate genes under biotic stress conditions.

**FIGURE 2:**
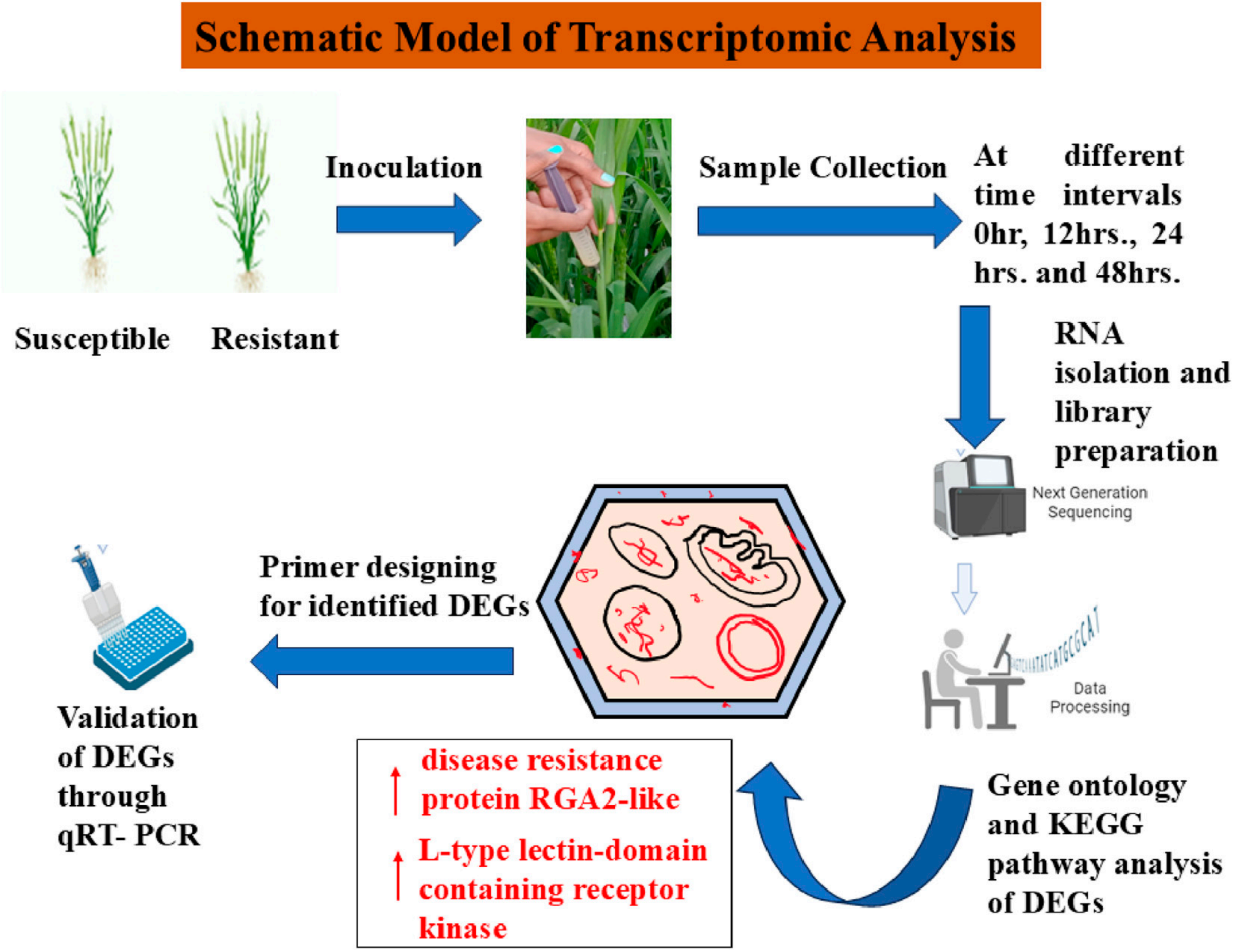
Schematic model of transcriptomic responses in resistant and susceptible genotypes. Retrieved from https://app.biorender.com/biorender-templates.

In Singh et al. (2024) conducted a transcriptomic study on the pathogenic dikaryophase to explore the function of the dikaryon in plant-pathogen interactions during the progression of KB. They analyzed the dikaryon (PSWKBGD-3) and its two monosporidial lines (PSWKBGH-1 and 2) using Illumina and PacBio sequencing, followed by annotation and comparative analysis of the three genomes to identify polymorphic SSR markers. At 24 h after inoculation (hai), 48 hai, and 7 days after inoculation (dai), a total of 54, 529, and 87 genes, respectively, were upregulated in the dikaryon stage. Additionally, 21, 35, and 134 genes of *T. indica* were activated exclusively in the dikaryon stage at these same time points. Furthermore, 23, 17, and 52 wheat genes were upregulated at 24 hai, 48 hai, and 7 dai, respectively, solely due to the presence of the dikaryon stage. To explore the molecular foundation of host-pathogen interactions, the transcriptomes of *T. indica*-inoculated wheat genotypes, both resistant (HD29) and susceptible (WH542), were examined. Over 80,000 genes were expressed in both types of wheat. Among these, 76,088 genes were expressed in both genotypes, with 3,184 genes significantly upregulated and 1,778 downregulated. Additionally, 4,113 genes were uniquely expressed in the susceptible genotype, while 5,604 were exclusive to the resistant genotype. Of these, 503 genes showed significant upregulation, and 387 were downregulated. The genes with the most significant differential expression were confirmed in both resistant and susceptible genotypes through qPCR analysis, showing similar expression levels as observed in RNA-Seq. Beyond the wheat, *T. indica* mapping accounted for 7.07% in resistant hosts and 7.63% in susceptible hosts upon infection, highlighting important pathogenesis-related genes. This pioneering study offered comprehensive insights into the wheat–*T. indica* interaction, aiding in the management of Karnal bunt disease in wheat (Gurjar et al., 2022). Figure 2 shows the schematic representation of transcriptomic analysis in resistant and susceptible genotypes.

Long non-coding RNAs (lncRNAs) are non-coding RNAs that exceed 200 nucleotides (Kim and Sung, 2012) and play a role in regulating cellular processes such as transcription, post-translational processing, chromatin modification, and gene expression (Isin and Dalay, 2015). They influence downstream target gene expression through molecular mechanisms at both transcriptional and post-transcriptional levels (Wang et al., 2019). Despite their limited protein-coding capacity, lncRNAs exert control over target gene expression during transcription and translation. Acting as molecular decoys, lncRNAs sequester miRNAs, thereby preventing their interaction with target messenger RNAs (Wang and Chang, 2011). Through these miRNA interactions, lncRNAs regulate various biological processes (Dhanoa et al., 2018). Although predicting targets remains challenging due to limited understanding of lncRNA-target interfaces, genome targeting and high-throughput screening underscore their vital role in stress tolerance (Gao et al., 2020). The moment plant experiences a stress, PAMP-triggered immunity (PTI) is activated by stress signals, leading to the generation of signaling molecules such as ROS. When pathogen virulence factors infiltrate plant cells, NB-LRR resistance genes initiate pathogen-specific effector-triggered immunity (ETI). Both PTI and ETI activate defense pathways. Long non-coding RNAs (lncRNAs) play a role in regulating plant defense by serving as miRNA precursors or target mimics. These non-coding RNAs enhance tolerance to biotic stress and modulate gene expression during plant pathogen infections, such as wheat powdery mildew. Studies have uravelled the contribution of lncRNAs in modulating the gene expression during host-pathogen interactions and in plant disease resistance such as powdery mildew (Cao et al., 2023), stripe rust (Das et al., 2023), leaf rust (Jain et al., 2020), Fusarium head blight in wheat (Duan et al., 2020). However, no study has been carried out to unveil the potential role of lncRNAs to decipher the molecular mechanism of resistance to Karnal bunt. Research on lncRNA unveiling disease resistance mechanisms needs to be explored for Kanal bunt of wheat.

## Proteomics

Proteomics complements genomics and transcriptomics by concentrating on gene products, offering a more direct perspective on cellular immunological processes. It allows for the simultaneous examination of protein localization, protein–protein interactions, enzymatic complexes, and post-translational modifications, all of which are crucial for understanding plant–pathogen interactions. (Pandey and Mann, 2000; Yates et al., 2009; Delaunois et al., 2014). Initially, ‘proteomics’ was a term used to describe techniques for analyzing multiple proteins at once. Over time, its definition has expanded to include any method that provides insights into the abundance, characteristics, interactions, activities, or structures of proteins within a sample. Currently, proteomics, particularly when based on mass spectrometry (MS), has evolved into a powerful “hypothesis-generating engine” that translates extensive data sets to elucidate complex biological processes (Meissner and Mann, 2014; Altelaar et al., 2013; Cravatt et al., 2007). In wheat, extensive proteomic research employing high-throughput techniques have been investigated on plant-pathogen interactions in response to *Fusarium graminearum,* the causal agent of Fusarium Head Blight (FHB) by (Fabre et al., 2021; Buchanan et al., 2025). In rusts fungi this omics technology is also utilized to identify novel biomarkers and disease resistance proteins (Yang et al., 2016a for stripe rust; Rampitsch et al., 2019; Song et al., 2011 for leaf rust). Nonetheless, literature on Karnal bunt resistance is limited. In 2018, Pandey et al. presented the first comprehensive proteome map through a comparative proteomic analysis of *T. indica* isolates with varying levels of virulence/aggressiveness. This study aimed to identify putative pathogenicity or virulence-related proteins expressed in the highly virulent isolate. The identified proteins associated with pathogenicity/virulence play crucial roles in stress response, degradation of the host cell wall, adhesion, penetration, invasion, colonization, activation of signal transduction pathways, and morphogenesis. In their He et al. (2022) identified differentially expressed proteins (DEPs), by employing iTRAQ and Ultra-High-Performance Liquid Chromatography (UHPLC)-MS/MS analyses. This analysis revealed 4,553 DEPs after inoculation with *Tilletia controversa* and 804 DEPs after inoculation with *Tilletia foetida*. Among these, 4,100 and 447 DEPs were upregulated, respectively, and were associated with metabolic processes, catalytic activity, photosynthetic membranes, transferase activity, and oxidoreductase activity. Table 3 summarizes the proteomic studies across major wheat pathogens. These studies open a pandora’s box to explore the new avenues for the identification of putative biomarkers for Karnal bunt resistance in wheat.

**TABLE 3:** Proteomic studies across major wheat pathogens and their key findings.

Pathogen	Key proteomic findings	Wheat response focus	Study highlights	References
*Tilletia indica* (Karnal bunt)	Identification of virulence factors including proteins for host defense suppression, lignin degradation, ROS generation, hydrolytic enzymes	Pathogen secretome and mycelial proteins	Integration of proteomics and genomics to identify candidate pathogenicity factors of *T. indica*	Pandey et al. (2018)
*Puccinia striiformis* (Stripe rust)	Differentially abundant proteins involved in transcription regulation, defense, ROS metabolism, splicing, chaperonins	Proteins linked to resistance gene loci, defense regulation	Transcriptome and proteome combined analysis identifying stress stage-specific modules and key regulators	Yang et al. (2016b)
*Fusarium graminearum* (Fusarium head blight)	Core wheat proteome changes under infection, fungal effectors correlated with wheat protein responses	Susceptibility factors, chloroplast function related proteins	Extensive proteome showing changes independent of fungal strain aggressiveness, dual proteomics approach	Fabre et al. (2021)
Other pathogens (leaf rust, powdery mildew)	Identification of proteins related to immunity and resistance pathways	Host immunity, stress response proteins	Proteomic profiling provides insights into resistance mechanisms at seedling and grain stages	Li et al., 2018;

## Metabolomics

Metabolomics examines small endogenous molecules such as sugars, amino acids, organic acids, and nucleotides to investigate the links between genetic structure, gene expression, protein function, and environmental influences (Fiehn, 2002). These molecules act as cellular substrates and byproducts, affecting phenotypes. This approach aids in clarifying the biochemical roles of crude proteins and their transformation into phenotypes. Plants have evolved defense mechanisms against pathogens, which include both constitutive and inducible defenses, as well as biochemical compounds and metabolites (Heuberger et al., 2014; Nielsen and Larsen, 2015). Since metabolites are products of transcription and translation, variations in their quantities reflect plant-microbe interactions and regulate defense in fungus-infected plants. Metabolomics has been utilized in wheat research to reveal stress tolerance mechanisms and identify candidate genes by correlating metabolite accumulation with stress responses. Its ability to complement other omics technologies makes it applicable across various organisms. As products of translation and transcription, metabolites play a vital role in plant-microbe interactions.

Plants have developed both constitutive and inducible defense against pathogens (Scheel, 1998). Variations in metabolites signal plant-microbe interactions and regulate defense mechanisms in infected plants. In wheat research, metabolomics has been employed to uncover mechanisms of stress tolerance and pinpoint candidate genes by linking metabolite accumulation with stress responses. Its capacity to complement other omics technologies makes it applicable to a wide range of organisms. As products of translation and transcription, metabolites are crucial in plant-microbe interactions. This field merges analytical chemistry with data processing to evaluate changes in metabolites in response to environmental factors.

Common platforms for metabolite analysis include mass spectrometry, NMR, LC-MS, and GC-MS. The sensitivity of mass spectrometry facilitates the exploration of the metabolome and the discovery of biomarkers. Advances in air pressure chemical ionization (APCI), ESI, and MALDI-TOF have enhanced the accuracy of mass spectrometry (Issaq et al., 2009). NMR is used to identify ligand properties and protein binding, while GC-MS analyzes volatile compounds. LC-MS employs ESI and APCI for analyzing higher mass metabolites. Untargeted metabolomics examines all detectable metabolites, whereas targeted approaches concentrate on specific categories. Data processing platforms such as MET-COFEA, MET-Align, ChromaTOF, and MET-XAlign handle high-throughput data sets (Ma et al., 2016; Misra and van der Hooft, 2016; Perez de Souza et al., 2017). The processing involves baseline correction, alignment, peak separation, and normalization prior to identification. Metabolome databases like METLIN, NIST, and GOLM are used for metabolite identification (Johnson et al., 2015). Tools like MetaboAnalyst 5.0, Cytoscape 3.10.1, and various statistical methods are employed to analyze data and detect metabolites (Tsugawa et al., 2015; Xie et al., 2015). These analyses help in identifying metabolic markers associated with different traits. The Plant Metabolic Network (PMN) and Metabolomics Workbench offer centralized databases of plant metabolites and pathways to support research.

Metabolomics profiling uncovers alterations in plant metabolites during pathogen infection (Allwood et al., 2008). Plants utilize a variety of defense mechanisms to combat pathogens. Decoding a plant’s entire metabolome is challenging due to the diversity of metabolites (Tenenboim and Brotman, 2016). Plants generate metabolites that function as biomarkers for resistance to biotic stress (Balmer et al., 2013). Comparative metabolic profiling of diseased and healthy plants reveals the metabolic networks involved in plant-pathogen interactions (López-Gresa et al., 2010; Ren et al., 2021 employed LC/MS metabolomics to investigate the changes in *T. controversa*-infected and noninfected grains. The analysis revealed an increase in prostaglandins and 9-hydroxyoctadecadienoic acids in the infected grains. The concentrations of cucurbic acid and octadecatrienoic acid were altered post-infection, impacting plant defense mechanisms. Eight metabolic pathways were activated during the pathogen-plant interactions, including those related to phenylalanine, isoquinoline alkaloid, starch and sucrose, tyrosine, sphingolipid, arginine and proline, alanine, aspartate, glutamate, and tryptophan metabolism. Similiarly Weed et al. (2021) explored the resource allocation of *Tilletia caries* from wheat throughout its lifecycle. Utilizing GC-TOF MS and UPLC tandem MS platforms, we discovered that *T. caries* has a minimal impact on the global metabolome of wheat but significantly alters key metabolites involved in nutrient uptake and diminishes host defense pathways. The findings highlighted metabolic traits useful for selecting *T. caries*-resistant wheat varieties for organic agriculture and identified metabolites that could aid in the early detection of infection. Table 4 summarizes the metabolomic profiling and identified key metabolites with respect to major wheat pathogens.

**TABLE 4:** Metabolomic studies for wheat fungal pathogens.

Pathogen	Key metabolomic findings	Wheat metabolite changes	Important metabolic pathways/Compounds	References
*Tilletia indica* (Karnal bunt)	Oxalic acid identified as a key pathogenicity factor	Alterations in organic acids and secondary metabolites	Involvement of oxalic acid and fungal toxin-related metabolites	Pandey et al. (2018)
*Puccinia striiformis* (Stripe rust)	Elevated flavonoid glycosides in resistant cultivars	Changes in lipids, fatty acids, phenylpropanoids	Phenylpropanoid biosynthesis, linoleic acid metabolism	Mashabela et al. (2022)
*Fusarium graminearum* (Fusarium head blight)	Differential accumulation of amino acids and hydroxycinnamate derivatives	Activation of shikimate-mediated secondary metabolism	Amino acid metabolism, tricarboxylic acid cycle	Liu et al., 2022; Dong et al., 2023
*Tilletia caries*	Metabolic traits resistance breeding and early detection	Metabolites linked to nutrient uptake and host defense	Significant alterations in nutrient uptake metabolites and defense pathways	Weed et al. (2021)

Metabolome, being smaller than the proteome and genome, allows for more straightforward data processing. Approximately 3,000 metabolites are involved in major metabolic pathways. The knowledge of metabolic QTLs (mQTLs) concerning metabolic networks holds promise for metabolomics-assisted breeding, aimed at developing elite cultivars and enhancing our understanding of quantitative genetics (Wen et al., 2015). Metabolic profiling facilitates the identification of SNP markers or mQTL mapping for candidate gene discovery by linking genotype to phenotype. Metabolic markers are instrumental in identifying agronomic traits and exploring the metabolic mechanisms underlying phenotypes (Fernandez et al., 2016). The mQTLs technique combines gene expression and metabolite profiles to connect phenotype with genotype (Wen et al., 2015). With the advent of next-generation sequencing (NGS), mQTLs for candidate genes can be identified using ultra-high-density maps (Scossa et al., 2016). The integration of multi-omics technologies with genetic methodologies enables the identification of genes that influence secondary metabolite production (Beleggia et al., 2016). Research has pinpointed mQTLs that regulate biotic interactions in plants. Modern sequencing techniques have facilitated genome sequencing and mQTL analyses in crops. Host-pathogen genes are identified through mQTL mapping, which analyzes plant resistance mechanisms. By integrating metabolomic data with other omics approaches and systems biology models, a comprehensive understanding of metabolite interactions within cellular networks can be achieved (Manickam et al., 2023).

## Multi-omics or integrated omics approach

The integration of genomics, transcriptomics, proteomics, and metabolomics has significantly enhanced our comprehension for plant disease resistance mechanisms. With the progress in omics technologies and computational tools, multi-omics approaches have become indispensable for tackling stress biology questions in crops and reducing false positives from single data sources (Ritchie et al., 2015; Subramanian et al., 2020). This interdisciplinary strategy has shed light on the molecular, genetic, and biochemical networks that play a role in host defense, facilitating the discovery of new resistance genes, pathways, and markers crucial for breeding initiatives. Web tools like PAINTOMICS, KaPPA-view, COVAIN, and O-miner are employed to analyze multi-omics datasets (Kuo et al., 2013; García-Alcalde et al., 2011; Tokimatsu et al., 2005; Sun and Weckwerth, 2012; Sangaralingam et al., 2019; Sehgal et al., 2023). PAINTOMICS allows for the integrated visualization of transcriptomics and metabolomics on KEGG pathway maps, while KaPPA-view merges transcript and metabolite data on metabolic pathway maps. COVAIN provides statistical analysis through KEGG pathway and gene ontology (Sun and Weckwerth, 2012).

Pandey et al., 2018 conducted the first integrated omics study by combining the genomics, proteomics and metabolomics dataset. They explored the proteomes of *T. indica* isolates with high (TiK) and low (TiP) virulence. In the TiK proteome, twenty-one protein spots that were upregulated were identified using MALDI-TOF/TOF. The sequences were found to be similar to fungal proteins crucial for plant infection, such as those involved in stress response, adhesion, penetration, colonization, and degradation of the host cell wall. By integrating these findings with the *T. indica* genome, homologs of pathogenicity proteins were identified. Malate dehydrogenase, found in TiK, facilitates the conversion of malate to oxaloacetate, which is a precursor to oxalic acid, a significant pathogenicity factor. GC-MS metabolic profiling confirmed these findings, revealing that oxalic acid was present only in the TiK isolate. This comprehensive approach identified pathogenicity factors, offering insights into fungal mechanisms and strategies for disease management.Similarly, in another study performed by the same group of researchers, the TiK isolate of *T. indica* was cultivated with a host factor derived from the developing wheat spikes of WH-542 to investigate the mechanisms of disease pathogenesis. Protein profiles from both mycelial and secreted proteins were examined using 2-DE, revealing fifteen and twenty-nine upregulated spots in mycelial and secreted proteins, respectively, which were identified through MALDI TOF/TOF. These proteins are involved in suppressing host defense, breaking down lignin, aiding pathogen adhesion, generating reactive oxygen species (ROS), producing hydrolytic enzymes, and detoxifying ROS. By integrating proteomic and genomic analyses, candidate pathogenicity factors were identified and functionally annotated through sequence and structure-based analysis, leading to the discovery of new virulence factors in *T. indica* (Pandey et al., 2019). The identification of markers and candidate genes through multi-omics accelerates the development of Karnal bunt-resistant wheat varieties. On the other hand, in rice (Rajamuthu et al., 2025) and legumes (Mohamedikbal et al., 2025), multi-omics studies have revealed important resistance genes, protein modifications and metabolic pathways, enabling precision breeding and improved disease management strategies.

By integrating genomics, transcriptomics, proteomics, and metabolomics data, multi-omics reveals key genes, proteins, enzymes, and metabolites that mediate pathogen infection and plant defense pathways. The integrative biology system paves way for the following approaches.

i) These mechanistic insights enable researchers to identify vulnerability nodes in pathogens, such as essential metabolic enzymes or detoxification pathways, or stress-response networks in plants, which serve as attractive targets for customized agrochemical interventions (Fan et al., 2025).
ii) Multi-omics aids in identifying novel virulence factors, resistance genes, and metabolic pathways unique to pathogens, guiding the design of fungicides with increased specificity and reduced non-target effects. For instance, proteomics and metabolomics data can highlight distinct enzymes, transporters, and regulatory proteins in fungal pathogens that are absent in crops, thereby minimizing phytotoxicity risks and optimizing the mode of action of new chemical agents (Rosli et al., 2024; Zhang et al., 2022).
iii) The integrative systems biology leads to systems-level understanding of biological processes and interactions by combining computer modeling with a variety of high-throughput multi-omics data. By combining information from several layers, makes it possible to find new connections and mechanisms and providing hypotheses for experimental validation.
iv) By simulating and forecasting biological system behavior under various contexts, predictive systems biology can guide targeted interventions.
v) These studies open new avenues to reveal the effectiveness of combining multiple omics datasets with computational tools to decipher complex host-pathogen interactions.

These approaches can be adapted for KB to enable targeted breeding, molecular diagnostics and precision disease management strategies unique to Karnal bunt. Wheat breeders now have access to a range of molecular tools and validated resistance sources, which aids in disease management and supports global trade by reducing dependence on chemical controls and easing quarantine restrictions.

## Multiepiomics

Epigenetics implies the heritable changes in the way of gene expression as a consequence of the modification of DNA bases, histone proteins, and/or non-coding-RNA biogenesis without disturbing the underlying nucleotide sequence. The changes occurring between DNA and its surrounding chromatin without altering its DNA sequence and leading to significant changes in the genome of any organism are called epigenetic changes (Shilpa et al., 2022).

Multi-epiomics uncovers the transcriptomic, proteomic, and metabolomic reactions during pathogen invasion, highlighting defense-related genes and epigenetic changes that influence resistance outcomes. These methods shed light on the co-evolution of hosts and pathogens, aiding in the development of sustainable resistance strategies. The integration of epigenome and transcriptome now makes it feasible to target susceptibility genes and modify regulatory elements. By considering the epigenomic context, engineering immune receptors, such as NLR proteins, facilitates the creation of synthetic immune defenses against pathogens. Research is centered on stable epigenetic alterations for enduring disease resistance, resulting in epigenetic memory in crops. Altering epigenetic patterns boosts resistance traits across generations (Miglani and Kaur, 2024).

In wheat, plants edited for *Mlo* genes have been developed. In hexaploid bread wheat, the complete loss of all three *Mlo* homologs (*TaMLO-A1, TaMLO-B1,* and *TaMLO-D1*) resulted in resistance to *B. graminis* f. sp. *tritici* (*Bgt*) (Wang et al., 2014; Li et al., 2022). The wheat gene *TaPsIPK1* was identified as an S gene, and its inactivation *via* CRISPR/Cas9 conferred resistance to wheat stripe rust (Wang et al., 2022). EDR1 is conserved across plant species (Frye et al., 2001). CRISPR/Cas9-generated *Taedr1* wheat plants, targeting all EDR1 homoeologs, exhibited resistance to *Bgt* without experiencing mildew-induced cell death (Zhang et al., 2017; Yin and Qiu, 2019). At the intersection of epigenomics, epitranscriptomics, and epiproteomics, there is an opportunity to explore post-translational modifications (PTLMs or PTMs) in detail, which modulate protein function and cellular processes. PTLMs, such as phosphorylation, acetylation, ubiquitination, and methylation, dynamically regulate protein activity, stability, localization, and interactions within the cell. These approaches deepen our understanding of the complex regulatory networks that govern cellular functions and pathways molecular mechanisms underlying complex traits and phenotypic plasticity hence paving the way for crop improvement and stress resilience.

## Artificial intelligence (AI) and machine learning in disease resistance

Artificial Intelligence involves computer systems performing tasks associated with human intelligence like learning and decision-making. Machine Learning (ML), an AI branch, creates algorithms enabling computers to perform tasks without explicit programming. Deep Learning (DL), a specialized field within ML uses neural networks with multiple layers to extract hierarchical features automatically. Machine Learning is a burgeoning field that significantly improves the performance and interpretation of trait associations. With technological advancements, larger datasets have become available for trait association studies. Machine Learning techniques can pinpoint genes that influence traits like disease resistance and forecast the effectiveness of these genes in defending against plant pathogens, thereby uncovering the interactions between plants and pathogens (Sperschneider, 2020).

Imaging platforms at ground, aerial and spatial levels collect images for plant phenotyping and stress detection (Sawant, 2017). Sensors capture spectral variation through RGB optical, infrared thermal, multispectral and hyperspectral sensors to detect disease. RGB cameras capture 400–750 nm wavelength in visible light, being most popular due to low cost (Li et al., 2014). Infrared thermal devices capture 3–5 μm or 7–14 μm spectral variation to detect temperature changes from disease infection (Zhu et al., 2018). Multispectral and hyperspectral cameras (Scotter, 2005) capture broader wavelengths for health assessments, detecting invisible symptoms through specific leaf component separation such as pigment changes (Wan et al., 2022), nutrient accumulation (de Oliveira et al., 2022) and other stressors (Guerrero et al., 2023). ML model detection accuracy depends on sensor and data collection methods chosen such as: scientific requirements for tracking early or late-stage symptoms; plant and pathogen species determining pixel resolution and wavelength needs; (c) environmental conditions affecting camera calibration and background complexity; and (d) available resources for implementing an effective data collection pipeline at sufficient resolution for model learning.

Climate change has a profound effect on crop health and the patterns of diseases (Burdon and Zhan, 2020), complicating their detection. It modifies temperature, precipitation patterns, and disease-related factors, which may render historical models less reliable (Yang et al., 2023). The increased variability in weather due to climate change results in unpredictable disease outbreaks (Rosenzweig et al., 2001). It also influences the interactions between crops and pathogens (Raza and Bebber, 2022), as well as crop phenology and vulnerability to diseases (Piao et al., 2019; Jeger, 2022). These climate impacts necessitate changes in irrigation, fertilization, and pesticide application (González-Domínguez et al., 2020). To tackle the complexities introduced by climate change, disease detection models must incorporate multiple data sources and Machine Learning techniques, although further research is essential. A recent study investigated by (Anand et al., 2024) Machine Learning methods for Karnal bunt prediction using meteorological data from different periods - February, March, 15 February to 15 March and overall period from Department of Climate Change and Agricultural Meteorology, PAU, Ludhiana. For each period, different disease prediction models performed well. Random forest regression for February, support vector regression (SVR) for March, SVR and BLASSO for 15 February to 15 March period, and random forest for overall period outperformed other models. In addition to this, Hasan et al. (2018) presented a deep learning approach using a robust R-CNN model to accurately detect, count, and analyze wheat spikes for yield estimation. The model was trained on images across different growth stages and optimized for diverse field scenarios.

Research has been carried out on the application of models for disease detection. Goyal et al. (2021) introduced an automated method for classifying wheat diseases, utilizing deep learning-based image analysis focused on spikes and leaves. This model achieved an accuracy rate of 97.88%, surpassing VGG16 and ResNet50 in terms of precision, recall, and F-score metrics. The results indicate the model’s potential for effective wheat disease classification and crop quality evaluation. Picon et al. (2019) created a deep residual neural network algorithm using 8,178 images to identify septoria, tan spot, and rust under real-world conditions. Their results confirmed the algorithm’s effectiveness in the early detection of wheat diseases.

After a disease is identified, autonomous systems for managing crop diseases can address the issue by applying pesticides in a targeted manner (Shaikh et al., 2016) or utilizing other forms of treatment (Abioye et al., 2022). These systems operate through robotic mechanisms that act based on data from sensors and imaging. By reducing pesticide usage (Mesías-Ruiz et al., 2023) and limiting runoff into waterways, autonomous management lessens environmental impact. Nonetheless, the systems face challenges related to their cost and complexity (Bhat and Nen-Fu, 2021). The high expense of implementation (Gackstetter et al., 2023) and concerns over autonomous decision-making (Bhat and Nen-Fu, 2021) are significant issues. Although these systems have the potential to transform agriculture and enhance sustainability, it is essential to address the technical and ethical challenges they present. In order to detect subtle disease symptoms, Machine Learning can be combined with remote sensing and high-throughput phenotyping for early Karnal bunt detection. This is achieved by evaluating multispectral and hyperspectral pictures taken by sensors or UAVs (Unmanned Aerial Vehicles). To identify diseased plants early, Machine Learning techniques such as random forests and deep learning extract important traits from vast amount of intricate phenotypic data. Through this integration, wheat fields may be monitored quickly, non-destructively, and extensively, facilitating better resistance screening and prompt disease control. These methods improve crop health assessment and early KB detection accuracy and efficiency (Gill et al., 2022; Anand et al., 2024).

Utilizing Machine Learning and image detection systems for automated crop disease identification can enhance agricultural productivity, contributing to food security and economic stability. These technologies offer precise, real-time assessments of crop health. Nonetheless, their implementation in developing nations encounters obstacles such as restricted access to hardware, software, technical skills, and infrastructure support.

## Future directions

India has been producing record wheat for last few years with the annual production exceeding 100 million tonnes. With a huge potential for exports, Karnal bunt, a quarantine disease poses major threat for the trade (Trethowan et al., 2018). Karnal bunt has a high potential for re-emergence in endemic regions and poses a risk of spreading to new regions, thereby causing economic damage to global wheat production and trade. Controlling KB epidemics and preventing its spread are priorities in wheat research, with the deployment of resistant cultivars being crucial. Historically, developing KB-resistant varieties has been challenging due to limited resistance sources and the environmental effects on the expression of quantitative resistance. These challenges can be addressed by mapping KB-resistance genes within wheat gene pools and introgressing them into elite cultivars. Identifying novel resistance sources requires new marker systems and improved MAS for KB resistance. Research must focus on host-pathogen interactions, race specification, gene mapping, annotation, and genomic selection to develop KB-resistant wheat cultivars.

By integrating omics approaches, mathematical and GS models can predict plant performance under stress, enabling wheat breeders to select the best gene-trait combinations for improved productivity. Analyzing the genome, transcriptomes, proteome, and metabolome simultaneously is essential for developing effective wheat improvement programs. Integrating AI with omics data encompassing genomics, transcriptomics, proteomics, and metabolomics facilitates early detection of plant diseases by analyzing molecular profiles before symptoms become visible. The combination of AI-assisted omics with high-throughput phenotyping enables real-time monitoring of plant health by linking molecular responses to phenotypic traits, thereby enhancing our understanding of plant defense mechanisms. AI and omics data support predictive modelling of disease dynamics, with Machine Learning and deep learning models taking into account genetic, molecular, and environmental variables to forecast outbreaks. AI-assisted omics techniques can advance crop breeding programs for disease resistance by identifying genetic markers linked to defense mechanisms. AI-driven recommendations using omics data allow for precise pesticide application, minimizing environmental impact and costs. Future research should aim to understand plant responses to combined biotic and abiotic stress through AI-assisted omics techniques, unravelling the plant pathogen interactions. The integration of remote sensing with AI-assisted omics offers spatial and temporal data on plant health and environmental conditions, enabling precise interventions to bolster plant defense and reduce crop losses. Integrative multi-omics enhances our understanding of how pathogens develop resistance to existing agrochemicals by identifying epigenetic, transcriptomic, and metabolic adaptations that can be countered with new chemistries. This approach leads to sustainable crop protection strategies and ensures the long-term efficacy of fungicides, particularly when combined with phenotypic and environmental profiling. Mindful applications of latest emerging technologies could help in eradication of Karnal bunt disease.

## References

[B1] Abioye E. A. Hensel O. Esau T. J. Elijah O. Abidin M. S. Z. Ayobami A. S. A. (2022). Precision irrigation management using machine learning and digital farming solutions. AgriEngineering 4 (1), 70–103. 10.3390/agriengineering4010006

[B2] Adams M. D. Kelley J. M. Gocayne J. D. Dubnick M. Polymeropoulos M. H. Xiao H. (1991). Complementary DNA sequencing: expressed sequence tags and human genome project. Science 252 (5013), 1651–1656. 10.1126/science.2047873 2047873

[B3] Allwood J. W. Ellis D. I. Goodacre R. (2008). Metabolomic technologies and their application to the study of plants and plant-host interactions. Physiol. Plant 132 (2), 117–135. 10.1111/j.1399-3054.2007.01001.x 18251855

[B4] Altelaar A. F. Munoz J. Heck A. J. (2013). Next-generation proteomics: towards an integrative view of proteome dynamics. Nat. Rev. Genet. 14 (1), 35–48. 10.1038/nrg3356 23207911

[B5] Anand S. Sandhu S. K. Biswas B. Bala R. (2024). Comparative analysis of different Karnal bunt disease prediction models developed by Machine Learning techniques for Punjab conditions. Int. J. Biometeorol. 68 (9), 1799–1810. 10.1007/s00484-024-02707-4 38805068

[B6] Aujla S. S. Sharma I. Gill K. S. Kour V. (1985). *Neovossia indica* on wild species of wheat. Indian Phytopathol. 38 (1), 191–199.

[B7] Aujla S. S. Sharma I. Gill K. S. (1990). Morphologic and physiologic resistance in wheat to Karnal bunt. Plant Dis. Res. 5, 119–121.

[B180] Bag T. K. Singh D. V. Tomar S. M. S. (1999). Inheritance of Karnal bunt (*Neovossia indica*) resistance in some Indian wheat (*Triticum aestivum*) lines and cultivars. J. Genet. Breed. 53, 67–72.

[B8] Bains S. S. Dhaliwal H. S. (1989). Release of secondary sporidia of *Neovossia indica* from inoculated wheat spikes. Plant Soil 115, 83–87. 10.1007/bf02220697

[B9] Bala R. Sharma A. Kashyap P. L. Rana B. Bains N. S. Sharma I. (2016). Molecular mapping of QTLs for Karnal bunt resistance in six near isogenic (NILs) populations of bread wheat. Indian Phytopathol. 69, 242–246.

[B10] Balmer D. Flors V. Glauser G. Mauch-Mani B. (2013). Metabolomics of cereals under biotic stress: current knowledge and techniques. Front. Plant Sci. 4, 82. 10.3389/fpls.2013.00082 23630531 PMC3632780

[B11] Bansal R. Singh D. V. Joshi L. M. (1983). Germination of teliospores of Karnal bunt of wheat. Seed Res. 11 (2), 258–261.

[B12] Bayer P. E. Petereit J. Durant É. Monat C. Rouard M. Hu H. (2022). Wheat Panache: a pangenome graph database representing presence-absence variation across sixteen bread wheat genomes. Plant Genome 15 (3), e20221. 10.1002/tpg2.20221 35644986 PMC12806990

[B13] Beavis W. D. (1994). “The power and deceit of QTL experiments: lessons from comparative QTL studies,” in Proceedings of the forty-ninth annual corn and sorghum industry research conference (Washington, DC: American Seed Trade Association), 250–266.

[B14] Beleggia R. Rau D. Laidò G. Platani C. Nigro F. Fragasso M. (2016). Evolutionary metabolomics reveals domestication-associated changes in tetraploid wheat kernels. Mol. Biol. Evol. 33 (7), 1740–1753. 10.1093/molbev/msw050 27189559 PMC4915355

[B15] Bhat S. A. Nen-Fu H. (2021). Big data and ai revolution in precision agriculture: survey and challenges. Ieee Access 9 (2021), 110209–110222. 10.1109/access.2021.3102227

[B16] Bishnoi S. K. He X. Phuke R. M. Kashyap P. L. Alakonya A. Chhokar V. (2020). Karnal bunt: a re-emerging old foe of wheat. Front. Plant Sci. 11, 569057. 10.3389/fpls.2020.569057 33133115 PMC7550625

[B17] Biswas B. Dhaliwal L. K. Mann S. K. Kaur G. Kashyap P. L. (2013). A model-based approach for predicting Karnal bunt disease of wheat under Punjab conditions. J. Agrometeorol. 15 (2), 158–162.

[B18] Bonde M. R. Prescott J. M. Matsumoto T. T. Peterson G. L. (1987). Possible dissemination of teliospores of *Tilletia-indica* by the practice of burning wheat stubble. Phytopathology 77, 639.

[B19] Bonde M. R. Berner D. K. Nester S. E. Peterson G. L. Olsen M. W. Cunfer B. M. (2004a). Survival of *T. indica* teliospores in different soils. Plant Dis. 88, 316–324. 10.1094/PDIS.2004.88.4.316 30812608

[B20] Bonde M. R. Nester S. E. Olsen M. W. Berner D. K. (2004b). Survival of teliospores of *Tilletia indica* in Arizona field soils. Plant Dis. 88 (8), 804–810. 10.1094/PDIS.2004.88.8.804 30812506

[B21] Brar G. S. Fuentes-Dávila G. He X. Sansaloni C. P. Singh R. P. Singh P. K. (2018). Genetic mapping of resistance in hexaploid wheat for a quarantine disease: Karnal bunt. Front. Plant Sci. 9, 1497. 10.3389/fpls.2018.01497 30386358 PMC6198147

[B22] Brooks S. A. See D. R. Brown‐Guedira G. (2006). SNP‐based improvement of a microsatellite marker associated with Karnal bunt resistance in wheat. Crop Sci. 46 (4), 1467–1470. 10.2135/cropsci2005.05-0065

[B23] Buchanan R. Shaban K. Liu B. Chan N. Serajazari M. Geddes-McAlister J. (2025). Temporal wheat proteome remodeling by deoxynivalenol reveals novel detoxification signatures and strategies across cultivars. Mol. Cell Proteomics 24 (6), 100988. 10.1016/j.mcpro.2025.100988 40349921 PMC12221369

[B24] Burdon J. J. Zhan J. (2020). Climate change and disease in plant communities. PLoS Biol. 18 (11), e3000949. 10.1371/journal.pbio.3000949 33232314 PMC7685433

[B25] Cao P. Wang Y. Ma Z. Xu X. Ma D. Yang L. (2023). Genome-wide identification of long intergenic non-coding RNAs of responsive to powdery mildew stress in wheat (*Triticum aestivum*). Front. Plant Sci. 14, 1297580. 10.3389/fpls.2023.1297580 38078075 PMC10704164

[B26] Carris L. M. Castlebury L. A. Goates B. J. (2006). Nonsystemic bunt fungi—*Tilletia indica* and *T. horrida*: a review of history, systematics, and biology. Annu. Rev. Phytopathol. 44 (1), 113–133. 10.1146/annurev.phyto.44.070505.143402 16480336

[B27] Casneuf T. Van de Peer Y. Huber W. (2007). *In situ* analysis of cross-hybridisation on microarrays and the inference of expression correlation. BMC Bioinforma. 8, 461. 10.1186/1471-2105-8-461 18039370 PMC2213692

[B28] Cravatt B. F. Simon G. M. Yates J. R. (2007). The biological impact of mass-spectrometry-based proteomics. Nature 450 (7172), 991–1000. 10.1038/nature06525 18075578

[B29] Crick F. (1970). Central dogma of molecular biology. Nat 227 (5258), 561–563. 10.1038/227561a0 4913914

[B30] Das P. Grover M. Mishra D. C. Guha Majumdar S. Shree B. Kumar S. (2023). Genome-wide identification and characterization of *Puccinia striiformis*-responsive lncRNAs in *Triticum Aestivum* . Front. Plant Sci. 14, 1120898. 10.3389/fpls.2023.1120898 37650000 PMC10465180

[B31] de Oliveira K. M. Furlanetto R. H. Rodrigues M. dos Santos G. L. A. A. Reis A. S. Teixeira Crusiol L. G. (2022). Assessing phosphorus nutritional status in maize plants using leaf-based hyperspectral measurements and multivariate analysis. Int. J. Remote Sens. 43 (7), 2560–2580. 10.1080/01431161.2022.2064198

[B32] Delaunois B. Jeandet P. Clément C. Baillieul F. Dorey S. Cordelier S. (2014). Uncovering plant-pathogen crosstalk through apoplastic proteomic studies. Front. Plant Sci. 5, 249. 10.3389/fpls.2014.00249 24917874 PMC4042593

[B33] Dhaliwal H. S. (1989). Multiplication of secondary sporidia of *Tilletia indica* on soil and wheat leaves and spikes and incidence of Karnal bunt. Can. J. Bot. 67, 2387–2390. 10.1139/b89-304

[B34] Dhaliwal H. S. Navarrete-Maya R. Valdez J. (1988). Scanning electron microscope studies of penetration mechanism of *Tilletia indica* in wheat spikes. Rev. Mex. Fitopathol. 7, 150–155.

[B35] Dhanoa J. K. Sethi R. S. Verma R. Arora J. S. Mukhopadhyay C. S. (2018). Long non-coding RNA: its evolutionary relics and biological implications in mammals: a review. J. Anim. Sci. Technol. 60, 25. 10.1186/s40781-018-0183-7 30386629 PMC6201556

[B36] Dong Y. Xia X. Ahmad D. Wang Y. Zhang X. Wu L. (2023). Investigating the resistance mechanism of wheat varieties to *Fusarium* head blight using comparative metabolomics. Int. J. Mol. Sci. 24 (4), 3214. 10.3390/ijms24043214 36834625 PMC9960685

[B37] Duan X. Song X. Wang J. Zhou M. (2020). Genome-wide identification and characterization of *Fusarium graminearum*-responsive lncRNAs in *Triticum aestivum* . Genes 11 (10), 1135. 10.3390/genes11101135 32992604 PMC7601646

[B38] Duveiller E. Mezzalama M. (2009). Karnal bunt screening for resistance and distributing KB free seed (CIMMYT). Available online at: https://repository.cimmyt.org/xmlui/bitstream/handle/10883/1289/96166.pdf (Accessed June 10, 2025).

[B39] Emebiri L. Singh S. Tan M. K. Singh P. K. Fuentes-Dávila G. Ogbonnaya F. (2019). Unravelling the complex genetics of Karnal bunt (*Tilletia indica*) resistance in common wheat (*Triticum aestivum*) by genetic linkage and genome-wide association analyses. G3 9 (5), 1437–1447. 10.1534/g3.119.400103 30824480 PMC6505162

[B40] Fabre F. Urbach S. Roche S. Langin T. Bonhomme L. (2021). Proteomics-based data integration of wheat cultivars facing *Fusarium graminearum* strains revealed a core-responsive pattern controlling *Fusarium* head blight. Front. Plant Sci. 12, 644810. 10.3389/fpls.2021.644810 34135919 PMC8201412

[B41] Fan B. L. Chen L. H. Chen L. L. Guo H. (2025). Integrative multi-omics approaches for identifying and characterizing biological elements in crop traits: current progress and future prospects. Int. J. Mol. Sci. 26 (4), 1466. 10.3390/ijms26041466 40003933 PMC11855028

[B42] Fernandez O. Urrutia M. Bernillon S. Giauffret C. Tardieu F. Le Gouis J. (2016). Fortune telling: metabolic markers of plant performance. Metabolomics 12 (10), 158. 10.1007/s11306-016-1099-1 27729832 PMC5025497

[B43] Fiehn O. (2002). Metabolomics--the link between genotypes and phenotypes. Plant Mol. Biol. 48 (1-2), 155–171. 10.1023/a:1013713905833 11860207

[B44] Frye C. A. Tang D. Innes R. W. (2001). Negative regulation of defense responses in plants by a conserved MAPKK kinase. Proc. Natl. Acad. Sci. U. S. A. 98 (1), 373–378. 10.1073/pnas.011405198 11114160 PMC14597

[B45] Fuentes-Davila G. Rajaram S. Van-Ginkel M. Rodriguez-Ramos R. Abdalla O. Mujeeb-Kazi A. (1996). Artificial screening for resistance to *Tilletia indica* . Cereal Res. Commun. 24 (4), 469–475.

[B46] Fuentes‐Davila G. Rajaram S. Singh G. (1995). Inheritance of resistance to Karnal bunt (*Tilletia indica* Mitra) in bread wheat (*Triticum aestivum* L.). Plant Breed. 114 (3), 250–252. 10.1111/j.1439-0523.1995.tb00804.x

[B47] Gackstetter D. von Bloh M. Hannus V. Meyer S. T. Weisser W. Luksch C. (2023). Autonomous field management–an enabler of sustainable future in agriculture. Agric. Syst. 206, 103607. 10.1016/j.agsy.2023.103607

[B48] Gao F. Cai Y. Kapranov P. Xu D. (2020). Reverse-genetics studies of lncRNAs-what we have learnt and paths forward. Genome Biol. 21 (1), 93. 10.1186/s13059-020-01994-5 32290841 PMC7155256

[B49] García-Alcalde F. García-López F. Dopazo J. Conesa A. (2011). Paintomics: a web-based tool for the joint visualization of transcriptomics and metabolomics data. Bioinformatics 27 (1), 137–139. 10.1093/bioinformatics/btq594 21098431 PMC3008637

[B50] Gill T. Gill S. K. Saini D. K. Chopra Y. de Koff J. P. Sandhu K. S. (2022). A comprehensive review of high throughput phenotyping and machine learning for plant stress phenotyping. Phenomics 2 (3), 156–183. 10.1007/s43657-022-00048-z 36939773 PMC9590503

[B51] Goates B. J. (2010). Survival of secondary sporidia of floret infecting *Tilletia* species: implications for epidemiology. Phytopathology 100, 655–662. 10.1094/PHYTO-100-7-0655 20528183

[B52] Goates B. J. Jackson E. W. (2006). Susceptibility of wheat to *Tilletia indica* during stages of spike development. Phytopathology 96, 962–966. 10.1094/PHYTO-96-0962 18944051

[B53] Gogoi R. Singh D. V. Srivastava K. N. Tomar S. M. S. Srivastava K. D. (2002). Morphological and isozymic variations among Karnal bunt resistant and susceptible genotypes of wheat-a comparison. Acta Phytopathol. Entomol. Hung. 37, 99–108. 10.1556/aphyt.37.2002.1-3.11

[B54] González-Domínguez E. Fedele G. Salinari F. Rossi V. (2020). A general model for the effect of crop management on plant disease epidemics at different scales of complexity. Agronomy 10 (4), 462. 10.3390/agronomy10040462

[B55] Goyal L. Sharma C. M. Singh A. Singh P. K. (2021). Leaf and spike wheat disease detection and classification using an improved deep convolutional architecture. Inf. Med. Unlocked 25, 100642. 10.1016/j.imu.2021.100642

[B56] Guerrero J. I. Martín A. Parejo A. Larios D. F. Molina F. J. León C. (2023). A general-purpose distributed analytic platform based on edge computing and computational intelligence applied on smart grids. *Sensors* (*Basel*.) 23, 3845. 10.3390/s23083845 37112186 PMC10140943

[B57] Gupta V. He X. Kumar N. Fuentes-Davila G. Sharma R. K. Dreisigacker S. (2019). Genome wide association study of Karnal bunt resistance in a wheat germplasm collection from *Afghanistan* . Int. J. Mol. Sci. 20 (13), 3124. 10.3390/ijms20133124 31247965 PMC6651844

[B58] Gurjar M. S. Jogawat A. Kulshreshtha D. Sharma S. Gogoi R. Aggarwal R. (2016). Intraspecific variation in *T. indica* isolates a pathogen causing Karnal bunt of Wheat. Indian Phytopathol. 69, 352–356.

[B59] Gurjar M. S. Jain S. Aggarwal R. Saharan M. S. Kumar T. P. J. Kharbikar L. (2022). Transcriptome analysis of wheat-*Tilletia indica* interaction provides defense and pathogenesis-related genes. Plants 11 (22), 3061. 10.3390/plants11223061 36432790 PMC9698794

[B60] Hasan M. M. Chopin J. P. Laga H. Miklavcic S. J. (2018). Detection and analysis of wheat spikes using Convolutional Neural Networks. Plant Methods 14, 100. 10.1186/s13007-018-0366-8 30459822 PMC6236889

[B61] He T. Xu T. Muhae-Ud-Din G. Guo Q. Liu T. Chen W. (2022). ITRAQ-based proteomic analysis of wheat (*Triticum aestivum*) spikes in response to *Tilletia controversa* Kühn and *Tilletia foetida* Kühn infection, causal organisms of Dwarf bunt and common bunt of wheat. Biol. (Basel). 11 (6), 865. 10.3390/biology11060865 35741386 PMC9220156

[B62] Heuberger A. L. Robison F. M. Lyons S. M. Broeckling C. D. Prenni J. E. (2014). Evaluating plant immunity using mass spectrometry-based metabolomics workflows. Front. Plant Sci. 5, 291. 10.3389/fpls.2014.00291 25009545 PMC4068199

[B63] Hussain B. Akpınar B. A. Alaux M. Algharib A. M. Sehgal D. Ali Z. (2022). Capturing wheat phenotypes at the genome level. Front. Plant Sci. 13, 851079. 10.3389/fpls.2022.851079 35860541 PMC9289626

[B64] Isin M. Dalay N. (2015). LncRNAs and neoplasia. Clin. Chim. Acta 444, 280–288. 10.1016/j.cca.2015.02.046 25748036

[B65] Issaq H. J. Van Q. N. Waybright T. J. Muschik G. M. Veenstra T. D. (2009). Analytical and statistical approaches to metabolomics research. J. Sep. Sci. 32 (13), 2183–2199. 10.1002/jssc.200900152 19569098

[B66] Jain N. Sinha N. Krishna H. Singh P. K. Gautam T. Prasad P. (2020). A study of miRNAs and lncRNAs during Lr28-mediated resistance against leaf rust in wheat (*Triticum aestivum* L.). Physiol. Mol. Plant 112. 101552. 10.1016/j.pmpp.2020.101552

[B67] Jeger M. J. (2022). The impact of climate change on disease in wild plant populations and communities. Plant Pathol. 71 (1), 111–130. 10.1111/ppa.13434

[B68] Johnson C. H. Ivanisevic J. Benton H. P. Siuzdak G. (2015). Bioinformatics: the next frontier of metabolomics. Anal. Chem. 87 (1), 147–156. 10.1021/ac5040693 25389922 PMC4287838

[B69] Kashyap P. L. Kaur S. Sanghera G. S. Kang S. S. Pannu P. P. S. (2011). Novel methods for quarantine detection of Karnal bunt (*Tilletia indica*) of wheat. Elixir Agric. 31, 1873–1876.

[B70] Kaur M. Singh R. Kumar S. Mandhan R. P. Sharma I. (2015). Identification of QTL conferring Karnal bunt resistance in bread wheat. Indian J. Biotechnol. 15 (1), 34–38.

[B71] Kim E. D. Sung S. (2012). Long noncoding RNA: unveiling hidden layer of gene regulatory networks. Trends Plant Sci. 17 (1), 16–21. 10.1016/j.tplants.2011.10.008 22104407

[B72] Koua A. P. Siddiqui M. N. Heß K. Klag N. Kambona C. M. Duarte-Delgado D. (2024). Genome-wide dissection and haplotype analysis identified candidate loci for nitrogen use efficiency under drought conditions in winter wheat. Plant Genome 17 (1), e20394. 10.1002/tpg2.20394 37880495 PMC12806935

[B73] Kumar J. Nagarajan S. (1998). Role of flag leaf and spike emergence stage on the incidence of Karnal bunt in wheat. Plant Dis. 82 (12), 1368–1370. 10.1094/PDIS.1998.82.12.1368 30845471

[B185] Kumar S. Chawla V. Yadav N. R. Sharma I. Yadav P. K. Kumar S. (2015). Identification and validation of SSR markers for Karnal bunt (*Neovossia indica*) resistance in wheat (*Triticum aestivum*). Indian J. Agric. Sci. 85 (5), 712–717.

[B74] Kumar S. Mishra C. N. Gupta V. Singh R. Sharma I. (2016). Molecular characterization and yield evaluation of near isogenic line (NIL) of wheat cultivar PBW 343 developed for Karnal bunt resistance. Indian Phytopathol. 69, 119–123. Available online at: https://epubs.icar.org.in/index.php/IPPJ/article/view/58379.

[B75] Kumar S. Singroha G. Singh G. P. Sharma P. (2021). Karnal bunt of wheat: etiology, breeding and integrated management. Crop Prot. 139, 105376. 10.1016/j.cropro.2020.105376

[B76] Kuo T. C. Tian T. F. Tseng Y. J. (2013). 3Omics: a web-based systems biology tool for analysis, integration and visualization of human transcriptomic, proteomic and metabolomic data. BMC Syst. Biol. 7, 64. 10.1186/1752-0509-7-64 23875761 PMC3723580

[B77] Li L. Zhang Q. Huang D. (2014). A review of imaging techniques for plant phenotyping. Sensors (Basel) 14 (11), 20078–20111. 10.3390/s141120078 25347588 PMC4279472

[B78] Li J. Liu X. Yang X. Li Y. Wang C. He D. (2018). Proteomic analysis of the impacts of powdery mildew on wheat grain. Food Chem. 261, 30–35. 10.1016/j.foodchem.2018.04.024 29739597

[B79] Li S. Lin D. Zhang Y. Deng M. Chen Y. Lv B. (2022). Genome-edited powdery mildew resistance in wheat without growth penalties. Nature 602 (7897), 455–460. 10.1038/s41586-022-04395-9 35140403

[B80] Lisker A. Maurer A. Schmutzer T. Kazman E. Cöster H. Holzapfel J. (2022). A haplotype-based GWAS identified trait-improving QTL alleles controlling agronomic traits under contrasting nitrogen fertilization treatments in the MAGIC wheat population WM-800. Plants (Basel) 11 (24), 3508. 10.3390/plants11243508 36559621 PMC9784842

[B81] Liu C. Chen F. Liu L. Fan X. Liu H. Zeng D. (2022). The different metabolic responses of resistant and susceptible wheats to *Fusarium graminearum* inoculation. Metabolites 12 (8), 727. 10.3390/metabo12080727 36005599 PMC9413380

[B82] López-Gresa M. P. Maltese F. Bellés J. M. Conejero V. Kim H. K. Choi Y. H. (2010). Metabolic response of tomato leaves upon different plant-pathogen interactions. Phytochem. Anal. 21 (1), 89–94. 10.1002/pca.1179 19866456

[B83] Lorenz K. Cohen B. A. (2012). Small-and large-effect quantitative trait locus interactions underlie variation in yeast sporulation efficiency. Genetics 192 (3), 1123–1132. 10.1534/genetics.112.143107 22942125 PMC3522155

[B84] Ma X. Xia H. Liu Y. Wei H. Zheng X. Song C. (2016). Transcriptomic and metabolomic studies disclose key metabolism pathways contributing to well-maintained photosynthesis under the drought and the consequent drought-tolerance in rice. Front. Plant Sci. 7, 1886. 10.3389/fpls.2016.01886 28066455 PMC5174129

[B85] Ma S. Wang M. Wu J. Guo W. Chen Y. Li G. (2021). WheatOmics: a platform combining multiple omics data to accelerate functional genomics studies in wheat. Mol. Plant. 14, 1965–1968. 10.1016/j.molp.2021.10.006 34715393

[B86] Ma J. Zhang M. Lv W. Tang X. Zhao D. Wang L. (2022). Overexpression of TaSNAC4-3D in common wheat (*Triticum aestivum* L.) negatively regulates drought tolerance. Front. Plant Sci. 13, 945272. 10.3389/fpls.2022.945272 35860542 PMC9289557

[B87] Mackay T. F. (2001). Quantitative trait loci in *Drosophila* . Nat. Rev. Genet. 2 (1), 11–20. 10.1038/35047544 11253063

[B88] Manickam S. Rajagopalan V. R. Kambale R. Rajasekaran R. Kanagarajan S. Muthurajan R. (2023). Plant metabolomics: current initiatives and future prospects. Curr. Issues Mol. Biol. 45 (11), 8894–8906. 10.3390/cimb45110558 37998735 PMC10670879

[B89] Manickavelu A. Kawaura K. Imamura H. Mori M. Ogihara Y. (2011). Molecular mapping of quantitative trait loci for domestication traits and β-glucan content in a wheat recombinant inbred line population. Euphytica 177, 179–190. 10.1007/s10681-010-0217-9

[B90] Mashabela M. D. Piater L. A. Steenkamp P. A. Dubery I. A. Tugizimana F. Mhlongo M. I. (2022). Comparative metabolite profiling of wheat cultivars (*Triticum aestivum*) reveals signatory markers for resistance and susceptibility to stripe rust and Aluminium (Al^3+^) toxicity. Metabolites 12 (2), 98. 10.3390/metabo12020098 35208172 PMC8877665

[B91] Meissner F. Mann M. (2014). Quantitative shotgun proteomics: considerations for a high-quality workflow in immunology. Nat. Immunol. 15 (2), 112–117. 10.1038/ni.2781 24448568

[B92] Mesías-Ruiz G. A. Pérez-Ortiz M. Dorado J. de Castro A. I. Peña J. M. (2023). Boosting precision crop protection towards agriculture 5.0 *via* machine learning. Machine Learning and emerging technologies: a contextual review. Front. Plant Sci. 14, 1143326. 10.3389/fpls.2023.1143326 37056493 PMC10088868

[B93] Miglani G. S. Kaur M. (2024). Multi-omics and crop improvement for sustainable agriculture. In Omics and Genome Editing: Revolution in Crop Improvement for Sustainable Agriculture 15-35. Cham: Springer Nature Switzerland.

[B94] Misra B. B. van der Hooft J. J. (2016). Updates in metabolomics tools and resources: 2014–2015. Electrophoresis 37 (1), 86–110. 10.1002/elps.201500417 26464019

[B95] Mitra M. (1931). A new bunt on wheat in India. Ann. Appl. Biol. 18, 178–179. 10.1111/j.1744-7348.1931.tb02294.x

[B96] Mitra M. (1937). Studies on the stinking smut or bunt of wheat in India. Ind. J. Agric. Sci. 7, 459–477.

[B97] Mohamedikbal S. Al-Mamun H. A. Bestry M. S. Batley J. Edwards D. (2025). Integrating multi-omics and Machine Learning for disease resistance prediction in legumes. Theor. Appl. Genet. 138 (7), 163. 10.1007/s00122-025-04948-2 40579624 PMC12204941

[B98] Montenegro J. D. Golicz A. A. Bayer P. E. Hurgobin B. Lee H. Chan C. K. (2017). The pangenome of hexaploid bread wheat. Plant J. 90 (5), 1007–1013. 10.1111/tpj.13515 28231383

[B99] Morgunov A. Montoya J. Rajaram S. (1993). Genetic analysis of resistance to Karnal bunt (*Tilletia indica* (Mitra)) in bread wheat. Euphytica 74 (1), 41–46. 10.1007/BF00033765

[B100] Myles C. Wayne M. (2008). Quantitative trait locus (QTL) analysis. Nat. Sci. Educ. 1 (1), 208.

[B101] Nagarajan S. Aujla S. S. Nanda G. S. Sharma I. Goel L. B. Kumar J. (1997). Karnal bunt (*Tilletia indica*) of wheat-a review. Rev. Plant Pathol. 76, 1207–1214.

[B102] Nelson J. C. Autrique J. E. Fuentes‐Dávila G. Sorrells M. E. (1998). Chromosomal location of genes for resistance to Karnal bunt in wheat. Crop Sci. 38, 231–236. 10.2135/cropsci1998.0011183x003800010039x

[B103] Nielsen K. F. Larsen T. O. (2015). The importance of mass spectrometric dereplication in fungal secondary metabolite analysis. Front. Microbiol. 6, 71. 10.3389/fmicb.2015.00071 25741325 PMC4330896

[B104] Pandey A. Mann M. (2000). Proteomics to study genes and genomes. Nature 405 (6788), 837–846. 10.1038/35015709 10866210

[B105] Pandey V. Singh M. Pandey D. Kumar A. (2018). Integrated proteomics, genomics, metabolomics approaches reveal oxalic acid as pathogenicity factor in *Tilletia indica* inciting Karnal bunt disease of wheat. Sci. Rep. 8 (1), 7826. 10.1038/s41598-018-26257-z 29777151 PMC5959904

[B106] Pandey V. Gupta A. K. Singh M. Pandey D. Kumar A. (2019). Complementary proteomics, genomics approaches identifies potential pathogenicity/virulence factors in *Tilletia indica* induced under the influence of host factor. Sci. Rep. 9 (1), 553. 10.1038/s41598-018-37810-1 30679765 PMC6346058

[B107] Perez de Souza L. Naake T. Tohge T. Fernie A. R. (2017). From chromatogram to analyte to metabolite. How to pick horses for courses from the massive web resources for mass spectral plant metabolomics. Gigascience 6, 1–20. 10.1093/gigascience/gix037 28520864 PMC5499862

[B108] Piao S. Liu Q. Chen A. Janssens I. A. Fu Y. Dai J. (2019). Plant phenology and global climate change: current progresses and challenges. Glob. Chang. Biol. 25 (6), 1922–1940. 10.1111/gcb.14619 30884039

[B109] Picon A. Alvarez-Gila A. Seitz M. Ortiz-Barredo A. Echazarra J. Johannes A. (2019). Deep convolutional neural networks for mobile capture device-based crop disease classification in the wild. Comput. Electron. Agric. 161, 280–290. 10.1016/j.compag.2018.04.002

[B110] Prescott J. M. (1984). “Overview of CIMMYT’s Karnal bunt programme,” in Proceedings of the conference on Karnal bunt (Obregon, Sonora, Mexico: CIMMYT), 24.

[B111] Qian L. W. Hickey L. T. Stahl A. Werner C. R. Hayes B. Snowdon R. J. (2017). Exploring and Harnessing haplotype diversity to improve yield stability in crops. Front. Plant Sci. 8, 1534. 10.3389/fpls.2017.01534 28928764 PMC5591830

[B112] Radecka-Janusik M. Piechota U. Piaskowska D. Góral T. Czembor P. (2022). Evaluation of Fusarium head blight resistance effects by haplotype-based genome-wide association study in winter wheat lines derived by marker Backcrossing approach. Int. J. Mol. Sci. 23 (22), 14233. 10.3390/ijms232214233 36430711 PMC9695032

[B113] Rajamuthu R. Tamilselvan A. Pandian V. Sooriya Moorthy L. Vedachallam V. Dhandapani U. (2025). Translating multi-omics insights into rice disease management: integrative approaches for sustainable resistance. Front. Plant Sci. 138, 102694. 10.1016/j.pmpp.2025.102694

[B114] Rampitsch C. Huang M. Djuric-Cignaovic S. Wang X. Fernando U. (2019). Temporal quantitative changes in the resistant and susceptible wheat leaf apoplastic proteome during infection by wheat leaf rust (*Puccinia triticina*). Front. Plant Sci. 10, 1291. 10.3389/fpls.2019.01291 31708941 PMC6819374

[B115] Rasheed A. Hao Y. Xia X. Khan A. Xu Y. Varshney R. K. (2017). Crop breeding chips and genotyping platforms: progress, challenges, and perspectives. Mol. Plant. 10 (8), 1047–1064. 10.1016/j.molp.2017.06.008 28669791

[B116] Raza M. M. Bebber D. P. (2022). Climate change and plant pathogens. Curr. Opin. Microbiol. 70, 102233. 10.1016/j.mib.2022.102233 36370642

[B117] Ren Z. Fang M. Muhae-Ud-Din G. Gao H. Yang Y. Liu T. (2021). Metabolomics analysis of grains of wheat infected and noninfected with *Tilletia controversa* Kühn. Sci. Rep. 11 (1), 18876. 10.1038/s41598-021-98283-3 34556726 PMC8460654

[B118] Riccioni L. Inman A. Magnus H. A. Valvassori M. Porta-Puglia A. Conca G. (2008). Susceptibility of European bread and durum wheat cultivars to *Tilletia indica* . Plant Pathol. 57, 612–622. 10.1111/j.1365-3059.2008.01830.x

[B119] Ritchie M. D. Holzinger E. R. Li R. Pendergrass S. A. Kim D. (2015). Methods of integrating data to uncover genotype-phenotype interactions. Nat. Rev. Genet. 16 (2), 85–97. 10.1038/nrg3868 25582081

[B120] Rosenzweig C. Iglesius A. Yang X. B. Epstein P. R. Chivian E. (2001). Climate change and extreme weather events-Implications for food production, plant diseases, and pests. Glob. Change Hum. Health 2, 90–104. 10.1023/A:1015086831467

[B121] Rosli M. A. F. Syed Jaafar S. N. Azizan K. A. Yaakop S. Aizat W. M. (2024). Omics approaches to unravel insecticide resistance mechanism in *Bemisia tabaci* (Gennadius) (Hemiptera: Aleyrodidae). Peer J. 12, e17843. 10.7717/peerj.17843 39247549 PMC11380842

[B122] Sangaralingam A. Dayem-Ullah A. Z. Marzec J. Gadaleta E. Nagano A. Ross-Adams H. (2019). Multi-omic’ data analysis using O-miner. Brief. Bioinform 20 (1), 130–143. 10.1093/bib/bbx080 28981577 PMC6357557

[B123] Sawant S. (2017). “Development of biosensors from biopolymer com posites,” in Biopolymer composites in electronics. Editors Sadasivuni K. K. Ponnamma D. Kim J. Cabibihan J.-J. Al-Maadeed M. A. (Amsterdam: Elsevier), 353–383.

[B124] Scheel D. (1998). Resistance response physiology and signal transduction. Curr. Opin. Plant Biol. 1 (4), 305–310. 10.1016/1369-5266(88)80051-7 10066609

[B125] Scossa F. Brotman Y. de Abreu E. Lima F. Willmitzer L. Nikoloski Z. (2016). Genomics-based strategies for the use of natural variation in the improvement of crop metabolism. Plant Sci. 242, 47–64. 10.1016/j.plantsci.2015.05.021 26566824

[B126] Scotter C. N. G. (2005). “Infrared spectroscopy. Near-infrared,” in Encyclopedia of analytical science. Editors Worsfold P. Townshend A. Poole C. 2nd edition (Amsterdam, Netherlands: Elsevier), 415–426.

[B127] Sehgal S. K. (2006). Studies on incorporation of Karnal bunt resistance and productivity traits from Aegilops Tauschii Coss . Into Wheat (*Triticum Aestivum L*.). (doctoral dissertation). PAU, Ludhiana, India.

[B128] Sehgal D. Dhakate P. Ambreen H. Shaik K. H. B. Rathan N. D. Anusha N. M. (2023). Wheat omics: advancements and opportunities. Plants 12 (3), 426. 10.3390/plants12030426 36771512 PMC9919419

[B129] Shaikh D. A. Akshay G. G. Prashant A. C. Parmeshwar L. K. (2016). Intelligent autonomous farming robot with plant disease detection using image processing. Int. J. Adv. Sci. Comp. Eng. 5 (4), 1012–1016. 10.17148/IJARCCE.2016.54248

[B130] Sharma I. Bains N. S. Singh K. Nanda G. S. (2005). Additive genes at nine loci govern Karnal bunt resistance in a set of common wheat cultivars. Euphytica 142, 301–307. 10.1007/s10681-005-2436-z

[B131] Shendure J. (2008). The beginning of the end for microarrays? Nat. Methods 5 (7), 585–587. 10.1038/nmeth0708-585 18587314

[B132] Shilpa Thakur R. K. Prasad P. Bhardwaj S. C. Gangwar O. P. Kumar S. (2022). Epigenetics of wheat-rust interaction: an update. Planta 255 (2), 50. 10.1007/s00425-022-03829-y 35084577

[B133] Singh T. (1992). Components of resistance in wheat and Karnal bunt [Neovossia indica (Mitra) Mundkur]. Ph. D. Thesis. Ludhiana, India: Punjab Agricultural University.

[B134] Singh G. Rajaram S. Montoya J. Fuentes- Davila G. (1995a). Genetic analysis of Karnal bunt resistance in 14 Mexican bread–wheat genotypes. Plant Breed. 114, 439–441. 10.1111/j.1439-0523.1995.tb00827.x

[B135] Singh G. Rajaram S. Montoya J. Fuentes-Davila G. (1995b). Genetic analysis of resistance to Karnal bunt (*Tilletia indica*, Mitra) in bread wheat. Euphytica 81, 117–120. 10.1007/BF00025422

[B136] Singh H. Grewal T. S. Pannu P. P. S. Dhaliwal H. S. (1999). Genetics of resistance to Karnal bunt disease of wheat. Euphytica 105 (2), 125–131. 10.1023/A:1003425729370

[B137] Singh S. Brown-Guedira G. L. Grewal T. S. Dhaliwal H. S. Nelson J. C. Singh H. (2003). Mapping of a resistance gene effective against Karnal bunt pathogen of wheat. Theor. Appl. Genet. 106 (2), 287–292. 10.1007/s00122-002-1112-0 12596729

[B138] Singh S. Sharma I. Sehgal S. K. Bains N. S. Guo Z. Nelson J. C. (2007). Molecular mapping of QTLs for Karnal bunt resistance in two recombinant inbred populations of bread wheat. Theor. Appl. Genet. 116, 147–154. 10.1007/s00122-007-0654-6 17952401

[B139] Singh K. Sharma P. Jaiswal S. Mishra P. Maurya R. Muthusamy S. K. (2024). Genome and transcriptome based comparative analysis of *Tilletia indica* to decipher the causal genes for pathogenicity of Karnal bunt in wheat. BMC Plant Biol. 24 (1), 676. 10.1186/s12870-024-04959-z 39009989 PMC11251232

[B140] Singh J. Aggarwal R. Gurjar M. S. Sharma S. Jain S. Saharan M. S. (2020). Identification and expression analysis of pathogenicity-related genes in *Tilletia indica* inciting Karnal bunt of wheat. Australas. Plant Pathol. 10, 393–402. 10.1007/s13313-020-00711-x

[B141] Singh S. Sehgal D. Kumar S. Arif M. A. R. Vikram P. Sansaloni C. P. (2020). GWAS revealed a novel resistance locus on chromosome 4D for the quarantine disease Karnal bunt in diverse wheat pre-breeding germplasm. Sci. Rep. 10, 5999. 10.1038/s41598-020-62711-7 32265455 PMC7138846

[B142] Sirari A. Sharma I. Bains N. S. Raj B. Singh S. Bowden R. L. (2008). Genetics of Karnal bunt resistance in wheat: role of genetically homogenous *Tilletia indica* inoculum. Ind. J. Genet. Plant Breed. 68 (01), 10–14.

[B143] Song X. Rampitsch C. Soltani B. Mauthe W. Linning R. Banks T. (2011). Proteome analysis of wheat leaf rust fungus, Puccinia triticina, infection structures enriched for haustoria. Proteomics 11 (5), 944–963. 10.1002/pmic.201000014 21280219

[B144] Sperschneider J. (2020). Machine Learning in plant-pathogen interactions: empowering biological predictions from field scale to genome scale. New Phytol. 228 (1), 35–41. 10.1111/nph.15771 30834534

[B145] Subramanian I. Verma S. Kumar S. Jere A. Anamika K. (2020). Multi-omics data integration, interpretation, and its application. Bioinform. Biol. Insights 14, 1177932219899051. 10.1177/1177932219899051 32076369 PMC7003173

[B146] Sun X. Weckwerth W. (2012). COVAIN: a toolbox for uni- and multivariate statistics, time-series and correlation network analysis and inverse estimation of the differential Jacobian from metabolomics covariance data. Metabolomics 8, 81–93. 10.1007/s11306-012-0399-3

[B147] Sun C. Dong Z. Zhao L. Ren Y. Zhang N. Chen F. (2020). The Wheat 660K SNP array demonstrates great potential for marker‐assisted selection in polyploid wheat. Plant Biotechnol. J. 18 (6), 1354–1360. 10.1111/pbi.13361 32065714 PMC7206996

[B148] Swati S. Goel P. (2010). Allelic relationship among genes for resistance to Karnal bunt (*Tilletia indica*) in bread wheat. Indian Phytopathol. 63, 6–10.

[B149] Tan M. K. Brennan J. P. Wright D. Murray G. M. (2013). A review of the methodology to detect and identify Karnal bunt—a serious biosecurity threat. Australas. Plant Pathol. 42, 95–102. 10.1007/s13313-012-0176-9

[B150] Tenenboim H. Brotman Y. (2016). Omic relief for the biotically stressed: metabolomics of plant biotic interactions. Trends Plant Sci. 21 (9), 781–791. 10.1016/j.tplants.2016.04.009 27185334

[B181] Thudi M. Mascher M. Jayakodi M. (2025). Pangenome charts the genomic path for wheat improvement. Trends Plant Sci. 30 (7), 687–689. 10.1016/j.tplants.2025.03.002 40113550

[B151] Tiwari V. K. Saripalli G. Sharma P. K. Poland J. (2024). Wheat genomics: genomes, pangenomes, and beyond. Trends Genet. 40 (11), 982–992. 10.1016/j.tig.2024.07.004 39191555

[B152] Tokimatsu T. Sakurai N. Suzuki H. Ohta H. Nishitani K. Koyama T. (2005). KaPPA-view: a web-based analysis tool for integration of transcript and metabolite data on plant metabolic pathway maps. Plant Physiol. 138 (3), 1289–1300. 10.1104/pp.105.060525 16010003 PMC1176402

[B153] Trethowan R. Chatrath R. Tiwari R. Kumar S. Saharan M. S. Bains N. (2018). An analysis of wheat yield and adaptation in India. Field Crops Res. 219, 192–213. 10.1016/j.fcr.2018.01.021

[B154] Tsugawa H. Cajka T. Kind T. Ma Y. Higgins B. Ikeda K. (2015). MS-DIAL: data-independent MS/MS deconvolution for comprehensive metabolome analysis. Nat. Methods 12 (6), 523–526. 10.1038/nmeth.3393 25938372 PMC4449330

[B155] Tyagi B. S. Shoran J. Singh S. K. Saharan M. S. Kumar J. Singh G. (2010). Genetic analysis of resistance to Karnal bunt caused by *Neovossia indica* in bread wheat (*Triticum aestivum*). Indian J. Agric. Sci. 80 (11), 952–955. Available online at: https://epubs.icar.org.in/index.php/IJAgS/article/view/1721.

[B186] USDA APHIS (2007). Karnal Bunt Manual. Animal and Plant Health Inspection Service. Riverdale, MD: USDA. Available online at: https://www.aphis.usda.gov/sites/default/files/karnal-bunt-manual.pdf .

[B156] Utah Department of Agriculture and Food (1996). Karnal bunt quarantine information. Available online at: https://ag.utah.gov/wp-content/uploads/2019/06/Karnal-Blunt-Quarantine-Information.pdf (Accessed September 10, 2025).

[B157] Velculescu V. E. Zhang L. Vogelstein B. Kinzler K. W. (1995). Serial analysis of gene expression. Science 270 (5235), 484–487. 10.1126/science.270.5235.484 7570003

[B158] Villareal R. L. Fuentes-Davila G. Mujeeb-Kazi A. (1995). Synthetic hexaploids × *Triticum aestivum* advanced derivatives resistant to Karnal bunt (*Tilletia indica* Mitra). Cereal Res. Commun., 127–132. Available online at: https://www.jstor.org/stable/23783894.

[B159] Virdi R. Mavi G. S. Bala R. Sohu V. S. Bains N. S. Kaur D. J. (2016). Genetic characterization of resistance to stripe rust, leaf rust, Karnal bunt and cereal cyst nematode in a multiple disease resistant wheat stock W8627. Ind. J. Genet. Plant Breed. 76 (1), 40–46. 10.5958/0975-6906.2016.00006.7

[B160] Wan L. Zhou W. He Y. Wanger T. C. Cen H. (2022). Combining transfer learning and hyperspectral reflectance analysis to assess leaf nitrogen concentration across different plant species datasets. Remote Sens. Environ. 269, 112826. 10.1016/j.rse.2021.112826

[B161] Wang K. C. Chang H. Y. (2011). Molecular mechanisms of long noncoding RNAs. Mol. Cell 43 (6), 904–914. 10.1016/j.molcel.2011.08.018 21925379 PMC3199020

[B183] Wang N. Tang C. Fan X. He M. Gan P. Zhang S. (2022). Inactivation of a wheat protein kinase gene confers broad-spectrum resistance to rust fungi. Cell 185, 2961–2974.e19. 10.1016/j.cell.2022.06.027 35839760

[B162] Wang Z. Gerstein M. Snyder M. (2009). RNA-Seq: a revolutionary tool for transcriptomics. Nat. Rev. Genet. 10 (1), 57–63. 10.1038/nrg2484 19015660 PMC2949280

[B163] Wang Y. Cheng X. Shan Q. Zhang Y. Liu J. Gao C. (2014). Simultaneous editing of three homoeoalleles in hexaploid bread wheat confers heritable resistance to powdery mildew. Nat. Biotechnol. 32 (9), 947–951. 10.1038/nbt.2969 25038773

[B182] Wang X. Zhi P. Fan Q. Zhang M. Chang C. (2019). Wheat CHD3 protein TaCHR729 regulates the cuticular wax biosynthesis required for stimulating germination of *Blumeria graminis* f. sp. *tritici* . J. Exp. Bot. 70, 701–713. 10.1093/jxb/ery377 30364999

[B164] Warham E. J. (1987). Studies on Karnal bunt of wheat. Ph.D. Thesis: Aberystwyth, UK: University of Wales.

[B165] Warham E. J. (1988). Teliospore germination patterns in *Tilletia indica* . Trans. Br. Mycol. Soc. 90, 318–321. 10.1016/s0007-1536(88)80104-9

[B166] Warham E. J. (1990). A comparison of inoculation techniques for assessment of germplasm susceptibility to Karnal bunt (*Tilletia indica*) disease of wheat. Ann. Appl. Biol. 116 (1), 43–60. 10.1111/j.1744-7348.1990.tb06585.x

[B167] Weed R. A. Savchenko K. G. Lessin L. M. Carris L. M. Gang D. R. (2021). Untargeted metabolomic investigation of wheat infected with stinking smut *Tilletia caries* . Phytopathology 111 (12), 2343–2354. 10.1094/PHYTO-09-20-0383-R 34865506

[B168] Wen W. Li K. Alseekh S. Omranian N. Zhao L. Zhou Y. (2015). Genetic determinants of the network of primary metabolism and their relationships to plant performance in a maize recombinant inbred line population. Plant Cell 27 (7), 1839–1856. 10.1105/tpc.15.00208 26187921 PMC4531352

[B169] Xie W. Lv X. Ye L. Zhou P. Yu H. (2015). Construction of lycopene-overproducing S*accharomyces cerevisiae* by combining directed evolution and metabolic engineering. Metab. Eng. 30, 69–78. 10.1016/j.ymben.2015.04.009 25959020

[B170] Yang Y. Yu Y. Bi C. Kang Z. (2016a). Corrigendum: quantitative proteomics reveals the defense response of wheat against *Puccinia striiformis* f. sp. tritici. Sci. Rep. 6, 38464. Erratum for: Sci Rep. 2016 Sep 28;6:34261. 10.1038/srep34261. PMID: 28004734; PMCID: PMC5177906. 10.1038/srep38464 28004734 PMC5177906

[B171] Yang Y. Yu Y. Bi C. Kang Z. (2016b). Quantitative proteomics reveals the defense response of wheat against *Puccinia striiformis* f. sp. *tritici* . Sci. Rep. 6, 34261. 10.1038/srep34261 27678307 PMC5039691

[B172] Yang L. N. Ren M. Zhan J. (2023). Modeling plant diseases under climate change: evolutionary perspectives. Trends Plant Sci. 28 (5), 519–526. 10.1016/j.tplants.2022.12.011 36593138

[B173] Yates J. R. Ruse C. I. Nakorchevsky A. (2009). Proteomics by mass spectrometry: approaches, advances, and applications. Annu. Rev. Biomed. Eng. 11, 49–79. 10.1146/annurev-bioeng-061008-124934 19400705

[B174] Yin K. Qiu J. L. (2019). Genome editing for plant disease resistance: applications and perspectives. Philos. Trans. R. Soc. Lond. B. Biol. Sci. 374 (1767), 20180322. 10.1098/rstb.2018.0322 30967029 PMC6367152

[B175] Yuan Y. Scheben A. Chan C. K. K. Edwards D. (2017). Databases for wheat genomics and crop improvement. Wheat Biotechnol. *Methods Mol. Biol*. 1679, 277–291. 10.1007/978-1-4939-7337-8_18 28913808

[B176] Zhang Y. Bai Y. Wu G. Zou S. Chen Y. Gao C. (2017). Simultaneous modification of three homoeologs of *TaEDR1* by genome editing enhances powdery mildew resistance in wheat. Plant J. 91 (4), 714–724. 10.1111/tpj.13599 28502081

[B177] Zhang L. Dong C. Chen Z. Gui L. Chen C. Li D. (2021). WheatGmap: a comprehensive platform for wheat gene mapping and genomic studies. Mol. Plant. 14, 187–190. 10.1016/j.molp.2020.11.018 33271333

[B178] Zhang Y. Zhao M. Chen W. Yu H. Jia W. Pan H. (2022). Multi-omics techniques for analysis antifungal mechanisms of lipopeptides produced by *Bacillus velezensis* GS-1 against *Magnaporthe oryzae in vitro* . Int. J. Mol. Sci. 23 (7), 3762. 10.3390/ijms23073762 35409115 PMC8998706

[B179] Zhu W. Chen H. Ciechanowska I. Spaner D. (2018). Application of infrared thermal imaging for the rapid diagnosis of crop disease. IFAC-PapersOnLine 51 (17), 424–430. 10.1016/j.ifacol.2018.08.184

